# A customised target capture sequencing tool for molecular identification of *Aloe vera* and relatives

**DOI:** 10.1038/s41598-021-03300-0

**Published:** 2021-12-21

**Authors:** Yannick Woudstra, Juan Viruel, Martin Fritzsche, Thomas Bleazard, Ryan Mate, Caroline Howard, Nina Rønsted, Olwen M. Grace

**Affiliations:** 1 Royal Botanic Gardens, Kew, Surrey, TW9 3AE UK; 2 Natural History Museum Denmark, University of Copenhagen, Gothersgade 130, 1153 Copenhagen, Denmark; 3 National Institute of Biological Standards and Control, South Mimms, UK; 4 Wellcome Sanger Institute, Wellcome Trust Genome Campus, Hinxton, Saffron Walden, CB10 1RQ UK; 5 National Tropical Botanical Garden, 3530 Papalina Road, Kalaheo, HI 96741 USA

**Keywords:** Phylogenomics, Plant genetics, Genetic markers, Next-generation sequencing

## Abstract

Plant molecular identification studies have, until recently, been limited to the use of highly conserved markers from plastid and other organellar genomes, compromising resolution in highly diverse plant clades. Due to their higher evolutionary rates and reduced paralogy, low-copy nuclear genes overcome this limitation but are difficult to sequence with conventional methods and require high-quality input DNA. *Aloe vera* and its relatives in the Alooideae clade (Asphodelaceae, subfamily Asphodeloideae) are of economic interest for food and health products and have horticultural value. However, pressing conservation issues are increasing the need for a molecular identification tool to regulate the trade. With > 600 species and an origin of ± 15 million years ago, this predominantly African succulent plant clade is a diverse and taxonomically complex group for which low-copy nuclear genes would be desirable for accurate species discrimination. Unfortunately, with an average genome size of 16.76 pg, obtaining high coverage sequencing data for these genes would be prohibitively costly and computationally demanding. We used newly generated transcriptome data to design a customised RNA-bait panel targeting 189 low-copy nuclear genes in Alooideae. We demonstrate its efficacy in obtaining high-coverage sequence data for the target loci on Illumina sequencing platforms, including degraded DNA samples from museum specimens, with considerably improved phylogenetic resolution. This customised target capture sequencing protocol has the potential to confidently indicate phylogenetic relationships of *Aloe vera* and related species, as well as aid molecular identification applications.

## Introduction

DNA sequencing has revolutionised the understanding of the tree of life through the use of standardised genomic regions, DNA barcodes^1^ which can be used to distinguish plant species or clades. A unified two-locus DNA barcode for land plants, comprising plastid (*matK*, *rbcL*) and nuclear ribosomal (ITS) markers^2,3^ was selected for having sufficient molecular variation in the middle and highly conserved sequences on both extremities of the regions, allowing consistent recovery using PCR primers^4^. Widespread sequencing efforts resulted in a robust order- and family-level framework for angiosperms and a more stable classification system^5^, as well as forming a strong basis for molecular identification work^3^.

Nonetheless, the traditional DNA barcode is of limited use in plant groups which underwent recent and/or rapid speciation^3^, and/or frequent hybridisation^6^. There are two main reasons for a lack of resolution in these plant groups: (1) a lack of informative variations due to limited molecular sequence evolution between lineages^7^; and (2) the ubiquity of hybridisation and introgression events in the plant kingdom which cannot be traced in chloroplast genes due to unipaternal inheritance^6^. Examples of these are spread throughout the angiosperm tree of life^8^. For instance, in the Asteraceae (daisy) family—famous for its high rates of hybridisation and with up to 33,000 species the largest plant family in the world—intrafamilial relationships could not be resolved even with the use of 10 chloroplast markers^9^.

Low-copy nuclear (LCN) genes are promising alternatives for plant clades in which traditional DNA barcodes cannot be successfully applied. The higher rate of molecular evolution compared to organellar genomes, combined with low levels of paralogy, make LCN genes ideal candidates for improved phylogenetics^6^, as well as accurate molecular identification^10,11^. However, the complexity of plant genomes makes detection and recovery of these genes complicated. Plant genomes are characterised by abundant repetitive elements (bolstering up to 80% of genome content^12^) and gene duplications arising from whole-genome duplication events throughout the evolutionary history of the angiosperms^13^. Obtaining LCN genes from plants can therefore become a costly, laborious and frustrating effort.

Target capture sequencing^14^ is a cost-efficient way to obtain large (nuclear) datasets from plants by reducing the effective genomic library size, retaining only targeted sequences. Applications are numerous and have included species restoration programmes^15^, SARS-Cov-2 coinfection testing^16^, trait discovery^17^ and resolving taxonomically challenging groups^18^. In-solution hybrid-capture with RNA probes^19^ allows hundreds of nuclear loci to be enriched and amplified for high-coverage in high-throughput sequencing^20^.

In recent years, the technique has shown promise for use in molecular identification studies^21^ due to its applicability to large numbers of samples simultaneously (48–96 samples per reaction^22^), low DNA input requirement (≥ 6.25 ng^23^) and high enrichment success irrespective of DNA degradation levels^24–26^. Indeed, nuclear target enrichment sequencing has revolutionised plant phylogenomics ranging from angiosperm-wide universal applications^27^ to order-^28^, family-^29^, genus-^30^ or even species-specific^31^ approaches. The cost-efficient high-throughput capture of variable LCN sequences has already resolved the relationships in several clades characterised by rapid diversification such as *Asclepia*^20^, *Dioscorea*^30^, *Rubus*^32^ and *Cyperus*^33^.

Angiosperm-wide universal target capture tools (e.g., Angiosperm V1^34^ and Angiosperms353^27^) have improved phylogenomic resolution in several plant clades^35^. Whilst more affordable than clade-specific target capture tools, the LCN loci targeted by universal tools are relatively conserved genes, having been designed for resolving deeper nodes in the Angiosperm tree of life^35^, limiting its application to recently and/or rapidly diversified clades. Moreover, the recovery of genes using the Angiosperm353 panel is generally poor in Monocot clades (e.g., < 37% in *Cyperus*^33^), further limiting its use in clade-specific studies.

Here, we focus on the leaf-succulent plant genus *Aloe* L. (Asphodelaceae, subfamily Asphodeloideae) with high species diversity (> 600 species^36^), rapid radiation^37^, and large genome sizes^38^. A reliable identification tool is needed to support the burgeoning international trade in this group, because processed plant material is extremely difficult to identify when lacking diagnostic morphological characters. In addition, standing questions regarding its systematics need to be resolved with a robust phylogenomic framework. *Aloe* vera and several other species, some wild-harvested, are popular in food- and health products, cosmetics and as ornamental plants^39^. All species of *Aloe*, except *Aloe vera*, are regulated by the Convention on International Trade of Endangered Species (CITES)^40^. This is due to the difficulty of identifying plant material, particularly the leaves which are commonly used, and the threats posed by habitat loss and wild harvesting for horticulture^41^. The regulation of *Aloe* species in trade has implications for their conservation as well as opportunities to meet consumer demand for *Aloe*-derived products and ornamental plants^37^.

Traditional DNA barcoding techniques using organellar markers have had limited success^11,37,42^ with only 30% of *Aloe* specimens correctly identified using the ITS1 region^10^. Obtaining LCN genes would be a significant step forward but has so far been hindered by the large and complex genomes of *Aloe* species: 1C-values range from 8.10–35.95 (mean 16.76) pg^43^ (compared to the mean angiosperm genome size of 5.13 pg^44^), despite aloes being almost exclusively diploid^38,45^. For this reason, LCN genes would also be highly desirable to avoid issues related to expectedly abundant^12^ high-copy regions across the genome^13^ whilst providing the necessary higher rates of molecular sequence variation to distinguish between species of *Aloe*.

We present a clade-specific RNA-bait panel for *Aloe vera* relatives (Alooideae) suitable for target enrichment of LCN genes based on newly generated transcriptome sequences. We tested the sensitivity of the *Aloe* custom bait panel on DNA samples from plant material of varying ages and quality representing 24 species, including heavily degraded samples from herbarium specimens. We also tested the limits of taxonomic distance for this method by including all three subfamilies of Asphodelaceae. Phylogenetic analyses were used to evaluate the potential for this target capture approach for recovering accurate species relationships through comparison with previous phylogenetic studies in the Alooideae^37,46^. The method holds promise for important applications of molecular identification such as conservation law enforcement, trade monitoring and quality assurance in the *Aloe* industry.

## Results

### Reference transcriptomes

Three replicate transcriptomes were sequenced for each of the four species (*Aloe arborescens*, *Aloe buettneri*, *Aloe vera* and *Aloidendron barberae*) from high-quality RNA extracts for which no degradation was visible on the TapeStation results. Raw read output varied from 31,953,823 (*Aloidendron barberae* replicate 1) to 50,801,082 read pairs (*Aloe vera* replicate 2), with an average of 35,747,180. An average of 88.2% survived trimming and quality filtering (Table S1). Each replicate was assembled separately with an average of 118,263 transcripts (Table S1). For each species the replicate with the highest number of transcripts was selected for LCN loci selection.

### Custom *Aloe* bait panel design

In total, 904 putative single- to low-copy nuclear genes (exonic regions only) were identified using MarkerMiner^47^ (based on a list of Angiosperm-wide single-copy status genes^48^), with putative intron–exon boundaries indicated through alignment with the *Oryza sativa* genome, of which 304 were detected in all four species. Of these, 187 remained after removing loci containing exons < 80 bp long (to ensure RNA-bait compatability) and/or with < 20 SNPs per 1,000 bp sequence (to ensure variable target loci). Six additional loci were absent only in the *Aloidendron barberae* transcriptome, of which two met our filtering criteria described above, bringing the total number of loci to 189. The custom *Aloe* myBaits® panel designed by Arbor Biosciences comprised a total of 19,922 RNA probes, each 80 bp in length, to target a total of 1,029 exons (Table S2) comprising 350,347 bp.

### Target capture sequencing

The MiSeq run generated 62,383,297 sample-assigned reads (300 bp paired end) for 24 samples that passed the quality filtering step, with an average of 2,712,317 quality-filtered reads per sample. Slightly different pooling strategies and a different sequencing platform (Illumina HiSeq, 150 bp paired end, Macrogen Inc.) led to a similar number of quality-filtered reads per sample for *Hemerocallis* (2,639,965) and *Bulbine* (3,050,140) but many more (10,785,948) for *Xanthorrhoea* (Table 1).

**Table 1:** Target capture sequencing statistics per sample. Origin of sample is denoted with ‘S’ for silica-dried freshly harvested material, ‘H’ for herbarium specimen, ‘P’ for DNA extracts from samples used in previously published studies and ‘R’ for RNA from freshly harvested material. *: Sample sequenced in larger multiplex run on Illumina HiSeq platform as part of a separate study.

Sample	Phytogeographic region	Origin of sample	Ultrasonication time (s)	Reads After trimming	Reads mapped	% reads on target	Total Assembled Target exon length	SLCN Loci with sequence	% Target length recovered
*Aloe aageodonta*	Tropical East Africa	S	50	2,399,050	1,268,854	54.2	321,066	189	92.5
*Aloe bakeri*	Madagascar	P	50	2,416,438	1,285,339	53.1	329,211	188	94.8
*Aloe ballyi*	Tropical East Africa	S	50	1,602,898	773,633	48.3	334,326	188	96.3
*Aloe brandhamii*	Tropical East Africa	H	50	2,789,581	142,523	4.5	149,709	168	43.1
*Aloe comptonii*	Southern Africa	S	50	1,498,657	805,082	53.7	323,970	188	93.3
*Aloe distans*	Southern Africa	P	50	3,640,964	2,043,250	56.1	327,033	189	94.2
*Aloe erinacea*	Southern Africa (Namibia)	S	50	3,876,389	2,318,089	59.8	323,070	188	93.1
*Aloe ferox*	Southern Africa	S	50	2,368,852	1,263,235	53.3	327,576	189	94.4
*Aloe flexilifolia*	Tropical East Africa	S	50	2,788,225	1,454,756	52.2	327,432	189	94.3
*Aloe framesii*	Southern Africa	S	50	2,542,514	1,380,373	54.3	322,503	187	92.9
*Aloe greatheadii*	South Tropical Africa	S	50	2,358,342	1,209,120	51.3	321,351	189	92.6
*Aloe jucunda*	Horn of Africa	P	50	2,818,267	1,477,635	52.4	324,225	189	93.4
*Aloe juvenna*	Tropical East Africa	P	50	1,090,090	557,847	51.2	318,018	188	91.6
*Aloe lateritia*	Tropical East Africa	S	50	2,542,942	1,261,381	49.6	327,498	187	94.3
*Aloe macrocarpa*	Horn of Africa	S	50	1,512,167	723,207	47.8	322,302	189	92.8
*Aloe marlothii*	South Tropical Africa	P	50	3,514,483	1,922,219	54.7	329,751	189	95.0
*Aloe mcloughlinii*	Horn of Africa	S	50	2,773,518	1,520,473	54.8	326,418	189	94.0
*Aloe percrassa*	Horn of Africa	H	–	3,696,487	1,978,408	53.5	323,346	187	93.1
*Aloe succotrina*	Southern Africa	S	50	5,184,554	3,133,167	60.4	331,827	189	95.6
*Aloe suffulta*	South Tropical Africa	S	50	2,324,265	1,145,238	49.7	329,352	188	94.9
*Aloe vaombe*	Madagascar	P	50	3,163,131	1,712,967	54.2	313,962	188	90.4
*Aloe viguieri*	Madagascar	P	50	2,560,543	1,434,442	56.0	330,045	189	95.1
*Aloe yemenica*	Arabian Peninsula	S	50	2,920,940	1,561,211	53.4	326,742	188	94.1
*Aloiampelos ciliaris*	Southern Africa	F		3,279,922	1,790,419	54.6	311,208	189	89.6
Bulbine frutescens*	–	P	60	3,050,140	454,810	14.9	257,868	183	74.3
Xanthorrhoea preissii*	–	P	50	10,785,948	4,880,245	45.2	250,695	183	72.2
Hemerocallis flava*	–	P	60	2,639,965	504,220	19.1	165,225	152	47.6
*Aloe arborescens*	Southern Africa	R	–	–	–	–	344,044	189	–
*Aloe buettneri*	Western Africa	R	–	–	–	–	349,657	189	–
*Aloe vera*	Cultivation	R	–	–	–	–	350,347	189	–
*Aloidendron barberae*	Southern Africa	R	–	–	–	–	340,629	187	–
Average genus *Aloe*				2,712,317	1,407,498	51.2	317,858	187	91.6

The percentage of on-target reads varied from 4.5% (*Aloe brandhamii*) to 60.4% (*Aloe succotrina*). For the MiSeq (ingroup) samples, the average read coverage varied from 14.7 (locus #19) to 3227.0 (locus #2) with an average of 657.6 over all loci (Table S2). Average read coverage per sample varied from 64.5 (*Aloe brandhamii*) to 1415.4 (*Aloe succotrina*) (Table S3). For the HiSeq (outgroup) samples, between 14.9% (*Bulbine frutescens*) and 45.2% (*Xanthorrhoea preissii*) of reads were on-target and the average read coverage over all loci ranged from 89.7 (*Bulbine frutescens*) to 921.4 (*Xanthorrhoea preissii*).

Three loci (#2, #31 and #181) had particularly high average read coverage estimates (e.g., > 2000). One of these loci (#31) was identified as a potential paralog (“Phylogenomic estimation” section below). In loci #2 and #31, a high number of reads mapped to one region of the gene caused by a repetitive sequence in one of the reference sequences (identified by visualisation in Tablet). For locus #2 this is the region of 1,564–1,785 bp in the *Aloe vera* reference, and for locus #31 this is the region of 140–160 in *Aloidendron barberae*. In locus #2 the repetitive element is mostly restricted to a single clade including *Aloe vera*.

**Figure 1:**
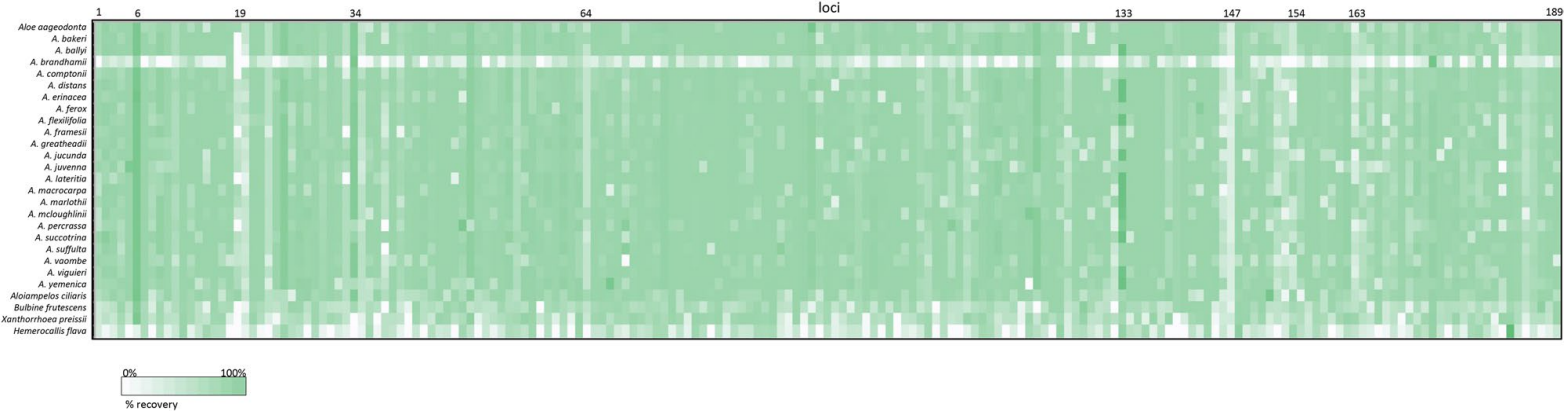
Heatmap indicating gene recovery success per gene in each sample, scale colour indicates success rate.

For ingroup samples, sequences were recovered for all loci in 12/24 enriched samples and only one sample (*Aloe brandhamii*, Table 1) was missing more than two loci (Fig. 1). An average of 93.6% of the total target length was recovered (Table 1) except for one sample derived from an herbarium specimen (43.1% *Aloe brandhamii*). Between the remaining *Aloe* samples, differences in target recovery were minimal, ranging from 90.4% (*Aloe vaombe*) to 95.6% (*Aloe succotrina*) (Table 1). For the related genus *Aloiampelos*, sequences were recovered for all loci and 89.6% of the total target length was assembled.

The average maximum sequence length recovered compared to the reference was 97.7% of the total length per locus; this dropped below 50% for only one locus, #147 (Table S2) for which the average recovered length was 41.6% of the reference length. Visualisation of the alignment revealed two large domains in this gene that were missing in nearly all enriched samples, which can be attributed to a lack of baits covering these regions. One locus was recovered in fewer than 21 samples (locus #19, 17/24 samples). For around a tenth of the loci (18 in total), HybPiper assembled more than 5% additional exon sequence which was particularly high (46.8%) in locus #133.

For the outgroup taxa the recovery rate was lower, ranging from 47.6% of the total target length in *Hemerocallis* to 74.3% in *Bulbine*. *Hemerocallis* had the lowest number of genes recovered (152, Table 1), whereas both *Xanthorrhoea* and *Bulbine* were both missing six loci, albeit different ones.

### Comparison with universal bait panels

A total of fifteen loci in our *Aloe* custom bait panel overlapped with the Angiosperms353^27^ universal bait panel, and a total of 27 with the Angiosperm V1^34^ tool (Table S2). Four of these loci were found in all three tools. All *Aloe* target loci were longer than the target loci in both universal panels. The *Aloe* bait panel targets a total surplus of 7,023 bases compared to the Angiosperms353 panel for overlapping loci, or 469 bases on average per locus.

Overall gene recovery rates for the overlapping loci were superior using the *Aloe* bait panel in all compared taxa, including the outgroup. For overlapping loci, the *Aloe* bait panel outperformed the Angiosperms353 panel by a factor of two for the ingroup taxa *Aloe marlothii* (95.9% of total target length recovered vs. 46.6%) and *Aloiampelos sp.* (90.9% vs. 44.0%) (Table S4). The total target recovery for *Aloidendron barberae* using the Angiosperms353 baits was slightly higher at 60.6%. One locus (#91 in the *Aloe* bait panel, #5660 in Angiosperms353) was not recovered in any ingroup taxa using the Angiosperms353 panel, whereas full-length recovery was achieved with the *Aloe* bait panel.

Recovery rate with the *Aloe* bait panel decreased with taxonomic distance, to 80.8% in *Bulbine frutescens* (subfamily Asphodeloideae), *Xanthorrhoea preissii* (subfamily Xanthorrhoeoideae, 79.2%) and *Hemerocallis flava* (subfamily Hemerocallidoideae, 61.6%). The *Aloe* bait panel outperformed the Angiosperms353 panel for *Bulbine frutescens* in overall gene recovery (80.8% vs. 49.1%) and performed similarly for *Xanthorrhoea preissii* (79.2% vs. 79.1%) and *Hemerocallis flava* (61.6% vs. 60.5%). For three different overlapping loci (#79, #100 and #182 in the *Aloe* bait panel; #6494, #5162 and #5859 in Angiosperms353), recovery was better with the Angiosperms353 panel in at least one of the outgroup taxa than with the *Aloe* bait panel (Table S4).

### Phylogenomic estimation

The supermatrix of 189 concatenated alignments, which included reference sequences from the four transcriptomes and sequences from the outgroup taxa, consisted of 374,466 bases, of which 265,106 remained after cleaning the alignment.

A dataset comprised of seven traditional markers (six chloroplast markers and nuclear ribosomal ITS) was compiled from 120 published sequences (“Phylogenetic estimation and comparison” section) obtained from GenBank and a further 25 sequences (13 *psbA*, five *rbcL*, four ITS, two *trnL-trnF* intergenic spacer and one *matK* sequences) which were added from assemblies using off-target reads in the present study (Table S5). Sequence length per marker ranged from 623 (*trnL* intron) to 1,566 (*matK*) bases in the traditional marker dataset and from 757 (locus #128) to 6353 (#57) bases in the LCN dataset. The total dataset for traditional markers comprised a concatenated supermatrix of 4,693 bases after cleaning the alignment (6,749 pre-cleaning) compared to 266,151 bases (373,705 pre-cleaning) in the LCN dataset.

**Figure 2:**
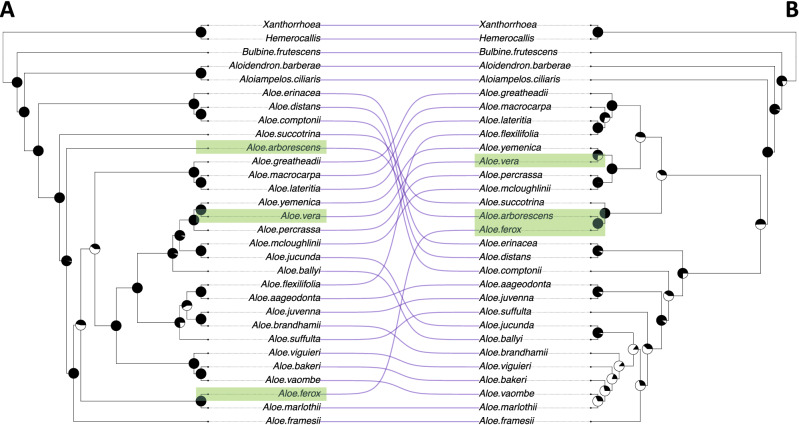
Cophylogeny (tanglegram) showing maximum-likelihood trees estimated with IQTree from 189 low-copy nuclear loci generated in this study (**A**) and from traditional markers (**B**). Pie charts indicate node support (black) calculated with bootstrap analysis (1000 replicates). Lines between the two phylogenies link tips belonging to the same taxon to indicate (dis)similarity between the topologies. Commercially used species are labelled in green in both topologies to highlight changes in relationships. For the taxa *Xanthorrhoea* and *Hemerocallis* only the genus name is indicated since different species were used in constructing the respective phylogenies (“Phylogenetic estimation and comparison” section for details).

Phylogenetic estimation using the two datasets produced different topologies (Fig. 2) with the only similarities being the sister relationship between *Aloe yemenica* and *A. vera*, and the relationships between *A. greatheadii*, *A. macrocarpa* and *A. lateritia*, although with higher support using the LCN dataset. Both topologies recovered *Bulbine* as sister to *Aloidendron*, *Aloiampelos* and *Aloe*. Only three out of eight sister relationships within *Aloe* were fully supported with the traditional dataset, whereas the LCN dataset produced full support for all of them.

**Figure 3:**
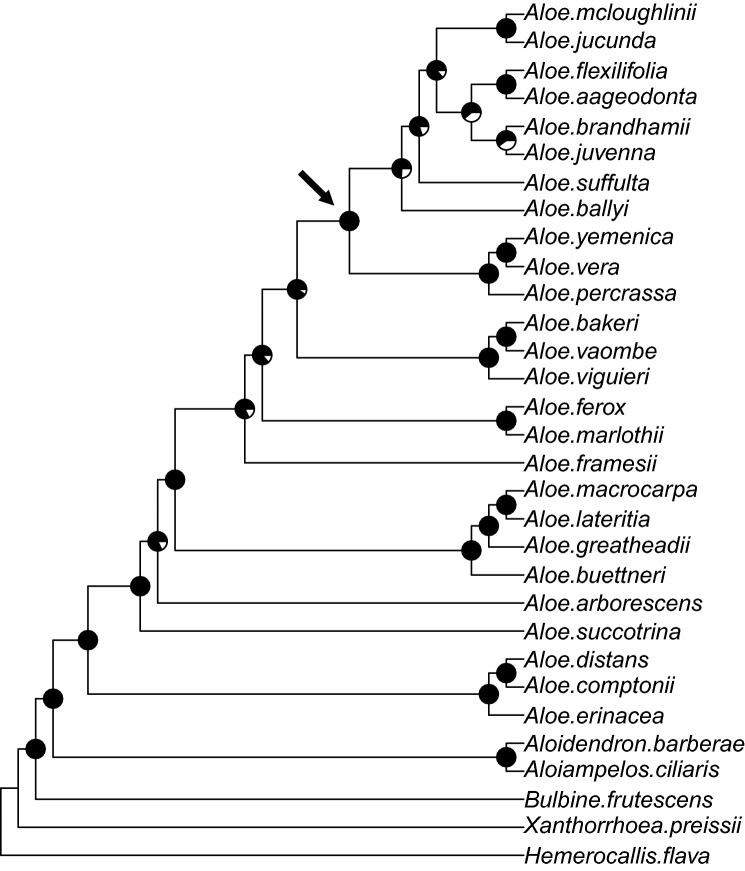
Phylogeny for *Aloe*, related genera and outgroups estimated with the coalescent-based ASTRAL-III algorithm from 188 maximum likelihood gene trees. Pie charts indicate node support (green) calculated as Local Posterior Probability by the ASTRAL software. Arrow indicates the node of the clade to which repetitive element of locus #2 is mostly restricted (“Target capture sequencing” section).

For the coalescent-based analysis, 189 gene trees were pruned to remove branches with bootstrap support values < 10. This resulted in the rejection of one gene tree (for locus #75) as the resulting pruned tree only consisted of one unresolved quartet. The remaining 188 pruned gene trees were summarised into a species tree (Fig. 3) using ASTRAL-III, where *Hemerocallis flava* was recovered as the most distant outgroup taxon from *Aloe*, followed by *Xanthorrhoea preissii* and *Bulbine frutescens*. All sister relationships except one (*Aloe juvenna*-*A. brandhamii*) were fully supported (LPP, Local Posterior Probability = 1.0). There was also full support for the separation of *Bulbine frutescens* from the ingroup taxa as well as for the monophyly of *Aloe*. Only three nodes had LPP values of < 0.80 and they all occurred in the ‘Tropical East African’ clade (Fig. 3), which included *Aloe brandhamii* (the only ingroup sample with recovery < 50%). More than 30% of quartets from the gene trees did not agree with the final species tree, with a normalised quartet score of 0.669.

Twenty-seven loci were identified as potential paralogs in at least one sample by the paralog warning script in HybPiper and seven additional loci were identified by manual inspection of the alignments. Paralogy was confirmed in 12 loci by visual inspection of unrooted gene trees generated in SplitsTree, Figure S6.

A separate analysis performed with the confirmed paralogous loci removed (177 loci) resulted in a single change in the topology regarding the sister relationship of *A. brandhamii*-*A. juvenna* (Figure S7). Using the full dataset, the two species are monophyletic in the topology supported by LPP = 0.60, whereas they are paraphyletic sister species in the reduced dataset supported by LPP = 0.42. In the rest of the topology, support increased slightly (LPP increase < 0.10) for 4 nodes and decreased slightly (LPP decrease < 0.05) for 2 nodes. For one particular node separating *A. framesii* from the remaining tree, support decreased significantly from LPP = 0.82 in the full dataset to LPP = 0.60 in the reduced dataset. A normalised quartet score of 0.676 indicated a slight decrease in gene tree discordance compared to the full dataset.

## Discussion

Consistently high recovery of 189 LCN genes with the *Aloe* custom target capture bait panel advances the possibilities for molecular identification and its applications in the trade and conservation of *Aloe vera* and related species. This is the first customised approach to sequence only LCN genes in *Aloe*^49^ and overcomes the challenges of variable, large and complex nuclear genomes encountered in this group^38,43^. It innovates on other high-throughput sequencing efforts, most notably whole chloroplast sequences^50^, which despite large volumes of data have had limited phylogenetic success^51^.

The *Aloe* custom bait panel compared favourably to custom bait panels for other genera, both in terms of enrichment efficiency, here evaluated as the proportion of on-target reads (e.g. 51.2% compared to 31.6% in *Dioscorea*^30^, 32.5% in *Asclepia*^20^), as well as average recovery rate (e.g. 91.5% compared to 78.6% in *Dioscorea* and 78.8% in *Asclepia*). With 74.3% of total target length recovered for 183/189 loci in *Bulbine*, the recovery in sister genera with the *Aloe* bait panel is also superior or comparable to that achieved in other custom bait panels, e.g., *Dioscorea* (24.2% of total target length in *Trichopus*)^30^ and *Asclepia* (81.3% in *Matelea*).

Other genera in the Alooideae, such as *Gasteria* and *Aloidendron*, are also potential targets for molecular identification given their value in (illegal) horticultural trade^52,53^. Target capture baits can be expected to perform on sequences with up to 30% divergence from the target^27^, expanding the potential application of a custom bait panel. The *Aloe* custom bait panel has purposefully been designed to be robust to the inclusion of closely related genera in the Alooideae subfamily^54^ by the inclusion of an *Aloidendron* transcriptome in the design process. This robustness was demonstrated by the high recovery rate for the genus *Aloiampelos* (89.6%, Table 1) and lower but nonetheless convincing recovery rates in other subfamilies of Asphodelaceae (72.2% in Xanthorrhoeoideae, 47.6% in Hemerocallidoideae), making this method suitable for phylogenomic studies in general related to *Aloe*. The decrease in recovery rates for the outgroup taxa follows taxonomic distance with *Bulbine frutescens* (subfamily Asphodeloideae) at 74.3%, *Xanthorrhoea preissii* (Xanthorrhoeoideae) at 72.2% and *Hemerocallis flava* (Hemerocallidoideae, the most distant subfamily from Alooideae) at 47.6%.

Historically, universal DNA barcodes were used for molecular identification studies^3^ but with the advent of target capture sequencing, these studies could benefit from clade-customised approaches yielding an increased amount of variable sequence data. The *Aloe* custom bait panel outperformed universal angiosperm bait panels^27,34^ (Table S4), highlighting the return on investment in developing a genus-focused custom bait panel for groups such as *Aloe* which have been particularly challenging subjects for phylogeneticists^36^. A snapshot comparison of two ingroup taxa (*Aloe marlothii* and *Aloiampelos* spp.) and three outgroup taxa (*Bulbine frutescens*, *Xanthorrhoea preissii* and *Hemerocallis flava*) that were target enriched using both the custom *Aloe* bait panel (this study) and the Angiosperms353^27^ approach (Grace et al., in review) was performed (Table S4). The 353 loci targeted in the Angiosperms353 bait panel^27^ are becoming the ‘standard’ loci for tree of life research on flowering plants^51^. However, the recovery rate is generally low (< 50%) for monocot plants: e.g., < 37% in *Cyperus*^33^, < 48% in *Gasteria* (174 genes ≥ 50%, Olivier Maurin, pers. comm.). The recovery rate for overlapping loci between the *Aloe* bait panel and the Angiosperms353 panel is < 50% in two thirds of samples, compared to > 90% using the *Aloe*-specific baits. Even for outgroup taxa, the *Aloe* custom bait panel performs better than the universal baits although this surplus decreases with taxonomic distance to *Aloe*. There seems to be a taxonomic ‘break-even point’ when moving to other subfamilies.

Historic and dried herbarium specimens have been described as ‘genomic treasure troves’^55^ due to their potential impact in studies of molecular systematics and this has been demonstrated in the Alooideae, too^49^. They can also provide a solid basis for molecular identification if type specimens were to be used to build a curated reference database. However, DNA from historical specimens is often degraded, especially when the plant tissue is dried slowly by heating at 60–70 °C^56^ as is the case for many succulent plant collections, and this has complicated recovery of nuclear genes in particular^55^. Target capture sequencing overcomes this burden by using small oligonucleotides to capture target DNAs in-solution^24,26^. Target recovery with the *Aloe* bait panel was unaffected using an herbarium specimen as source material (e.g., 93.1% in *Aloe percrassa*, Table 1) indicating the potential for this tool to be used on material with varying levels of DNA degradation, such as extracts from cosmetic or food products common to the *Aloe* industry.

The lower recovery in another herbarium specimen (*Aloe brandhamii*, 43.1%) is likely due to over-fragmentation of the DNA extract prior to library preparation. The sample was treated in the same way as high-molecular-weight samples in our pilot study which likely over-sheared the DNA fragments below the size selection range, thereby reducing the library complexity which in turn would limit the recovery of target gene sequences (Figure S9 for TapeStation electorphorogram of DNA extract). This example highlights the importance of modified fragmentation protocols on a sample-per-sample basis to optimise target recovery in target capture sequencing studies.

One of the main benefits of utilising nuclear loci is the potential for hundreds of independently evolving loci to be analysed individually as gene trees. This can potentially give many more independent molecular identification hypotheses than a single-locus approach using ITS would. It also allows for coalescent-based analyses that are more robust in inferring incomplete lineage sorting^57–59^, which can improve phylogenetic resolution. Our *Aloe* tree contains evidence of incomplete lineage sorting, as indicated by differences in gene tree topologies and a normalised ASTRAL quartet score of 0.669. Indeed, while the support for two deeper nodes in a maximum likelihood tree is < 50 (Fig. 2A), the ASTRAL summary tree is far better resolved (Fig. 3) and is consistently better resolved than a tree estimated from published sequences of 7 loci (Fig. 2B).

The 189 nuclear loci show a distinctive geographic pattern in the *Aloe* phylogeny, with geographical clades suggesting pulsed radiations and speciation events^37^. The clear separation between these well-defined clades in our study, as well as accurate discrimination on the species level (Fig. 3), suggest that our approach would be an excellent candidate for a molecular identification tool. A large reference database of > 300 species will be curated to apply the tool to realistic market samples as well as CITES-restricted plants.

## Conclusions

With the design of a novel RNA-bait panel for target capture sequencing, we presented here a significant leap towards accurate molecular identification in a rapidly diversified group of succulent plants, with large and complex genomes. A fully resolved phylogeny is important for further studies of *Aloe*. Considering the economic importance of species such as *Aloe vera* and *Aloe ferox*, there is a need for an updated DNA barcoding tool for control on quality assurance and international trafficking related to CITES^40^. The use of LCN genes in DNA barcoding was suggested several years ago^10,11^ and successful examples are emerging, such as for the medicinally important plant *Anacyclus pyrethrum*^21^. Our LCN framework adds to these, achieving high on-target ratios, high target recovery rates and excellent phylogenomic resolution. It significantly improves species discrimination and compares favourably to universal bait panels, justifying a customised approach for the Alooideae and opening the possibility for use as a barcoding tool.

## Methods

### Transcriptome (exome) sequencing

We sequenced the leaf transcriptomes of four species (Table S8 for accession information)—*Aloe vera* (L.) Burm.f., *A. arborescens* Mill., *A. buettneri* A.Berger and *Aloidendron barberae* (Dyer) Klopper & Gideon F.Sm.—to generate nuclear exonic data for bait design. The *Aloe* species were selected to represent the phylogenetic diversity found in the genus—as based on the most recently published comprehensive phylogeny^37^—to select polymorphic LCN genes for capture that will likely be resolutive for other *Aloe* spp. The *Aloidendron* species was included to ensure the downstream bait panel design would be efficient for enriching samples across the Alooideae clade in order to resolve outstanding questions of systematics in this group^54,60^. Leaves were harvested for RNA extraction from living plants at the Royal Botanic Gardens, Kew. All plants were sampled at 7 am on 08 August 2018. A single leaf of each plant was excised, and tissue samples of approximately 1 cm^2^, prepared from the isolated outer leaf mesophyll, were flash-frozen and stored on dry ice for two hours.

RNA was extracted from three replicates per species (c. 20 mg) using a Plant RNEasy kit (Qiagen, Hilden, Germany). The RNA extractions were subsequently treated with an Ambion TURBO DNA-free™ (ThermoFisher Scientific, Waltham, MA, USA) reagent kit to remove traces of DNA and divalent cations that can catalyse RNA degradation. The level of RNA degradation was assessed by capillary electrophoresis using an RNA 6000 Pico kit on a 2100 Bioanalyzer (Agilent, Santa Clara, CA, USA).

cDNA libraries were built using an EpMotion 5075t automatic liquid handler (Eppendorf, Hamburg, Germany) through a Poly-A capture-based method using a TruSeq™ Stranded mRNA Library preparation kit (Illumina, San Diego, CA, USA), Agencourt AMPure XP beads (Beckman Coulter, Brea, California, USA) for clean-up steps and a SuperScript™ II reverse transcriptase (ThermoFischer Scientific). Samples were indexed using 6 bp-long indexes from a TruSeq™ RNA Single Indexes Set B kit (Illumina). Indexed libraries were quantified using a Qubit 1 × dsDNA HS Assay Kit (ThermoFischer Scientific) on a Qubit 4 (ThermoFischer Scientific) fluorometer and the fragment size distribution was determined by capillary electrophoresis using a High-Sensitivity DNA Kit (Agilent) on a 2100 Bioanalyzer (Agilent). Pooled libraries were sequenced for 2 × 150 pairedend cycles with a High Output Kit v2 (Illumina) on a NextSeq 500 platform (Illumina). Raw reads were converted to fastq format with bcl2fastq version 2.17.1.14 (Illumina), checked for sequence quality using FastQC v0.11.7^61^ and MultiQC v1.0^62^, and trimmed to remove Illumina adapters and poor-quality bases with Cutadapt v1.16^63^ using a Phred score of 30 as threshold. Trimmed reads with < 50 bp length were excluded from the analysis. Transcripts were assembled de novo from the trimmed and filtered reads using Trinity v2.8.3^64^ and checked for quality indicators (number of Trinity transcripts and ‘genes’, GC-content, contig N50, mean and average contig length) using the TrinityStats script provided with the package.

### Custom bait panel design

We used MarkerMiner version 1.2^47^ to detect LCN genes present in the transcriptome assemblies based on a published set of LCN genes common to all angiosperms^48^. Intron–exon boundaries were identified by alignment with the fully annotated *Oryza sativa* v7 genome as reference^65^ using MAFFT^66^ as part of the MarkerMiner pipeline. Loci were selected from the MarkerMiner output based on presence at least in the transcriptomes of the three *Aloe* species. We used the local BLAST function in Geneious v8 (Biomatters, Auckland, New Zealand) against the missing transcriptome for those loci that were detected only in three transcriptomes, to add the missing reference transcript. To obtain the final set of loci for RNA-bait panel design, we removed loci containing mid-locus exons < 80 bp long, to avoid ambiguous RNA-baits, and loci with < 20 SNPs per 1,000 bp sequence length to ensure sufficient informative sites. Finally, we trimmed the alignments on both ends to ensure completely overlapping sequence alignments for improved versatility in the bait panel.

The target loci alignments were used to design a final custom panel of 19,922 RNA probes (“baits”) of 80 bases each for a myBaits Custom DNA-Seq kit produced by Arbor Biosciences (Ann Arbor, Michigan, USA) with 3 × tiling on average. The initial bait panel design was checked for non-overlap with high-copy loci such as plastid loci (based on publicly available *A. maculata* and *A. vera* plastomes^67^) and repetitive elements using RepeatMasker^68^ for simple repeats and monocot-specific elements. The bait panel design was further reduced by removing baits with either high levels of redundancy (e.g. > 95% identical sequence-overlap with 83% of probes’ sequence) or high melting-temperature (e.g. > 65 °C T_m_ or > 75% GC-content).

### Bait panel performance testing

The application of our bait panel design was tested in a target capture sequencing experiment with 23 species from the genus *Aloe* L. and one species of the closely related genus *Aloiampelos* Klopper & Gideon F.Sm (Table S8). The species were selected to represent infrageneric morpho-groups recognised in *Aloe*^69–71^ and major clades in a previously published phylogeny^37^. Samples of 18 species were obtained from plants of known wild provenance in the living collections of the Royal Botanic Gardens, Kew and two samples were collected from pressed specimens from the Kew Herbarium (K) of varying age. DNA extracts of eight additional samples were added from previous studies^37,46^ where fresh or silica-dried material was used from either natural populations or from the living collections at the Royal Botanic Gardens, Kew. These included specimens representing the three Asphodelaceae subfamilies: *Bulbine frutescens* (L.) Willd. (subfamily Asphodeloideae), *Xanthorrhoea preissii* Endl. (subfamily Xanthorrhoeoideae), *Hemerocallis flava* L. (subfamily Hemerocallidoideae).

A single leaf was harvested from the plant, the inner leaf mesophyll tissue removed, and the outer leaf mesophyll dried in silica gel for at least one week. DNA was subsequently extracted from approximately 20 mg dried tissue using a Plant DNEasy Kit (Qiagen).

Leaf material from pressed herbarium specimens was carefully excised from the sheet (approximately 20 mg) and DNA was extracted using a CTAB protocol^72^, in which DNA was precipated at −20 °C for one week, and cleaned using Agencourt AMPure XP beads (Beckman Coulter). The concentration of DNA in all total genomic DNA extracts was quantified using a Quantus™ fluorometer (Promega, Maddison, Wisconsin, USA) and fragment size distribution was determined on a 4200 TapeStation (Agilent).

High molecular-weight DNA samples (23 in total) were fragmented by ultra-sonication for 50 s. (peak power: 50; duty factor 20; 200 cycles/burst) using an M220 Focused ultrasonicator (Covaris, Woburn, Massachusetts, USA), Table 1 for details. DNA libraries were prepared from ± 100 ng input DNA with an average insert size of 570 bp using a NEBNext® Ultra™ II Library Prep Kit and using 8 bp dual indexes for multiplexed sequencing (NEBNext® Dual Index Primer Set 1, New England Biolabs, Ipswich, Massachusetts, USA) supplemented with Agencourt AMPure XP beads (Beckman Coulter) for size selection and cleaning steps following the provided protocol. Libraries were diluted to 10 nM according to DNA concentration, quantified using a Quantus fluorometer (Promega), and fragment size distribution, determined with a 2100 BioAnalyzer (Agilent) and pooled in equal quantities.

The concentrated pool of 24 libraries (± 550 ng DNA) was enriched with the custom *Aloe* myBaits Kit (Arbor BioSciences) during 24 h at a constant 65 °C, following the manufacturer’s protocol. Before sequencing, the enriched pool was amplified using 18 PCR cycles (45 s. extension time each) and universal P5 and P7 primers (New England Biolabs), following the settings from the myBaits protocol. The amplified libraries for our pilot study were sequenced in-house with 2 × 300 paired-end cycles using a MiSeq Reagent Kit v3 on a MiSeq platform (Illumina).

Sequences for the outgroup taxa were available from another study (Woudstra et al., unpublished), obtained using a similar protocol with the differences being the ultra-sonication time (60 s. instead of 50), the size of pools in the enrichment reaction (12 instead of 24) and the sequencing platform (Illumina HiSeq (2 × 150 bp) instead of MiSeq).

Raw Illumina paired-end reads were quality controlled by examining FastQC^61^ reports for per-base sequence quality, read length distribution and GC content, among other parameters. Illumina adaptors and poor-quality reads were removed with Trimmomatic v0.39^73^ using a Phred average quality score of 30 as a minimum threshold value to either discard reads or trim them from the 3’ end. Trimmed reads were assembled using HybPiper v1.2^74^ with the selected target sequences from the transcriptomes that were used in the bait panel design as a reference (“Custom bait panel design” section). The HybPiper stats script was used to determine the number of on-target reads per sample as well as sequence lengths of assembled exons per locus and per sample to calculate recovery statistics. Read coverage was calculated per gene and per sample by mapping filtered reads onto the reference sequences (results from HybPiper) used in the bait panel design and visualising this in Tablet v1.21.02.08^75^. Reads were mapped to each of the reference sequences individually and the number of reads reported per locus per sample is the highest number among the four (three for loci #188 and #189) reference sequences. Read coverage was then calculated as the number of reads multiplied by the read length (300 bp for MiSeq, 150 bp for HiSeq) and divided by the total length of the locus (based on the reference).

### Comparison with universal bait panels

The performance of our custom *Aloe* bait panel was evaluated by in silico comparison to two published universal Angiosperms353^27^ bait panels. Overlapping loci were identified using a local BLAST search in Geneious v8 (Biomatters) using the target reference file (available in the supplementary materials^27^) against the *Aloe* bait panel target reference. Two ingroup taxa, *Aloe marlothii* and *Aloiampelos* sp., as well as the three outgroup taxa were enriched and sequenced both with the *Aloe* bait panel and in another study using the Angiosperms353 panel (Grace et al., in preparation). Additionally, *Aloidendron barberae*, used in this study for transcriptome sequencing to serve as reference material in the bait panel design, was also enriched with the Angiosperms353 panel. A comparison of gene recovery rates between the two panels was performed for these taxa with loci containing > 5% sequence overlap.

For completeness, the *Aloe* bait panel was compared to the older universal Angiosperm V1 target enrichment toolkit^34^ by blasting it against the target reference file to determine overlapping loci.

### Phylogenetic estimation and comparison

Phylogenies were estimated from the low-copy nuclear (LCN) dataset generated in the present study, and traditional marker dataset from loci used in the most recently published phylogeny for *Aloe* and related genera^37^ for comparison. Sequences for the traditional dataset were obtained from GenBank, from previous studies by Grace et al.^37^ and Dee et al.^46^ (Table S5). Missing sequences from this dataset were (partly) filled in silico by assemblies with HybPiper v1.2^74^ using off-target reads from our pilot study and sequences used in Grace et al.^37^ as a reference. For the outgroup taxa representing subfamilies Xanthorrhoeoideae and Hemerocallidoideae, we did not find an exact species match in the Grace et al. reference^37^ with the samples used in our pilot study and therefore took available sequences from another member of these genera: e.g., *Xanthorrhoea resinosa* Pers. and *Hemerocallis littorea* Makino, respectively.

For the LCN dataset, sequences were combined with the target reference sequences from the transcriptomes to generate 189 alignments (exons-only) using MAFFT v7.450^66^.

For comparison with the traditional marker dataset, *Aloe buettneri* was excluded from the LCN dataset to ensure complete taxon overlap and alignments were concatenated using FASconCAT-G v1.04^76^. A total of seven alignments were produced from the traditional dataset and combined into a supermatrix using the ‘concatenate’ tool in Geneious v9 (Biomatters). Both supermatrix alignments were cleaned using trimAl v1.2^77^ using the ‘-automated1’ function and maximum-likelihood trees were estimated with IQTree v1.6.12^78^ under a general time reversible (GTR) model combined with a gamma-distribution for rate heterogeneity and a proportion of invariant sites. Bootstrap support values for the trees were estimated with 1000 replicates.

Both phylogenetic trees were rerooted at the node between *Hemerocallis* and *Xanthorrhoea* in R v4.0.3^79^ using the ‘ape’ package v5.4-1^80^ and compared in a tanglegram using the package ‘phytools’ v0.7-70^81^ with pie charts to visualise the support of the nodes. Scripts is available in Suppl. Mat. S10.

The full LCN dataset, comprising 31 taxa, was analysed in a coalescent-based model using ASTRAL-III^59^. This method determines gene tree discordance by counting the overlapping quartets between gene trees and the summary species tree to assess the level of incomplete lineage sorting. To this extent, maximum-likelihood gene trees were first estimated from the individual locus-alignments with IQTree v1.6.12^78^ using the specifications above and by estimating phylogenetic resolution in likelihood ratio test and bootstrap support values with 1000 replicates each. Branches with low support (BS < 10) were removed from the gene trees using the ‘nw_ed’ application from Newick-utilities v1.6^82^. A species tree was estimated and scored with ASTRAL v5.7.3^59^. The tree was visualised in R v5.4-1 using the phytools package v0.7-70, Suppl. Mat. S11 for script.

For paralogy assessment we used both the ‘paralog warning’ output of HybPiper and visual inspection of the alignments individually for misaligned sequences. Where paralogy was suspected, we estimated relationships between species for the alignment with SplitsTree v4.16^83^ to detect long branches that are indicative of paralogy. A separate ASTRAL-III analysis was performed on a dataset where the loci identified as paralogs were removed, using the same parameters as described above.

### Plant collection statements

All plant samples newly collected in this study were taken from existing specimens in the living collections at Royal Botanic Gardens, Kew. These collections fully comply with international legislation, including the Convention on Biological Diversity (CBD), the Convention on International Trade of Endangered Species (CITES) and the Nagoya Protocol for equitable sharing of benefits. Where DNA samples were taken from previous studies, the authors carefully checked that proper sample collection permits and agreements were in place at the time of the respective study, e.g., OM Grace et al., 2015, *BMC Evol. Biol.*; R Dee et al., 2018, *Bot. J. Lin. Soc.* The authors declare that the use of plant parts in this study fully complies with international, UK national and Royal Botanic Gardens, Kew institutional guidelines and legislation.

## Supplementary Information

Supplementary Information 1.

Supplementary Information 2.

Supplementary Information 3.

Supplementary Information 4.

Supplementary Information 5.

Supplementary Information 6.

Supplementary Information 7.

Supplementary Information 8.

Supplementary Information 9.

Supplementary Information 10.

Supplementary Information 11.

Supplementary Information 12.
