# Supplementary material for: A customised target capture sequencing tool for molecular identification of Aloe vera and relatives
Source: Sci Rep. 2021 Dec 21;11:24347. doi: 10.1038/s41598-021-03300-0 (PMC8692607; doi:10.1038/s41598-021-03300-0)
Supplement: Supplementary file 1 — Supplementary Information 1. [file 41598_2021_3300_MOESM1_ESM.docx]

[TITLE]: “A customised target capture sequencing tool for molecular identification of *Aloe vera* and relatives”

[SUPPLEMENTARY FILE S1]: Transcriptome assembly statistics

Table S1 Assembly statistics for each transcriptome sample. AAR = Aloe arborescens, ABA = Aloidendron Barberae, ABU = Aloe buettneri and AVE = Aloe vera. ^Read pairs after trimming. *Aloe vera transcriptome sequenced and assembled as part of the 1000 Plants Initiative project^1^ using SOAPdenovo assembly software. **Total number of contigs as determined from the available data. N50 = assembly quality as contiguity, sequence length of shortest contig at 50% of total transcript length.

| Sample | Read Pairs^ | Transcripts | %GC | Contig N50 | Assembled bases |
| --- | --- | --- | --- | --- | --- |
| AAR 1 | 28,033,977 | 72,073 | 46.21 | 1288 | 59,460,785 |
| AAR 2 | 30,750,672 | 91,599 | 45.41 | 1415 | 78,856,913 |
| AAR 3 | 29,033,906 | 100,687 | 45.04 | 1457 | 86,172,797 |
| ABA 1 | 28,042,817 | 107,486 | 45.17 | 1293 | 81,583,587 |
| ABA 2 | 31,971,886 | 102,529 | 45.58 | 1357 | 81,861,151 |
| ABA 3 | 28,244,323 | 124,096 | 44.57 | 1395 | 99,097,199 |
| ABU 1 | 38,451,894 | 149,129 | 44.39 | 1418 | 116,671,517 |
| ABU 2 | 30,532,013 | 116,097 | 45.08 | 1449 | 95,194,304 |
| ABU 3 | 31,245,902 | 150,634 | 44.72 | 1354 | 112,935,074 |
| AVE 1 | 30,880,289 | 128,070 | 45.79 | 1278 | 93,277,885 |
| AVE 2 | 43,408,903 | 165,405 | 44.85 | 1375 | 124,786,508 |
| AVE 3 | 27,606,112 | 111,354 | 45.74 | 1343 | 85,265,551 |
| AVE 1KP* | 8,109,163 | 49,983** | 45.48 | 1413 | 31,232,573 |

**Reference**

1 Carpenter, E. J. *et al.* Access to RNA-sequencing data from 1,173 plant species: The 1000 Plant transcriptomes initiative (1KP). *GigaScience* **8**, doi:10.1093/gigascience/giz126 (2019).
