# Supplementary material for: A customised target capture sequencing tool for molecular identification of Aloe vera and relatives
Source: Sci Rep. 2021 Dec 21;11:24347. doi: 10.1038/s41598-021-03300-0 (PMC8692607; doi:10.1038/s41598-021-03300-0)
Supplement: Supplementary file 6 — Supplementary Information 6. [file 41598_2021_3300_MOESM6_ESM.pdf]

[SUPPLEMENTARY FILE S6]: SplitsTree trees for paralogy indication

Locus #12

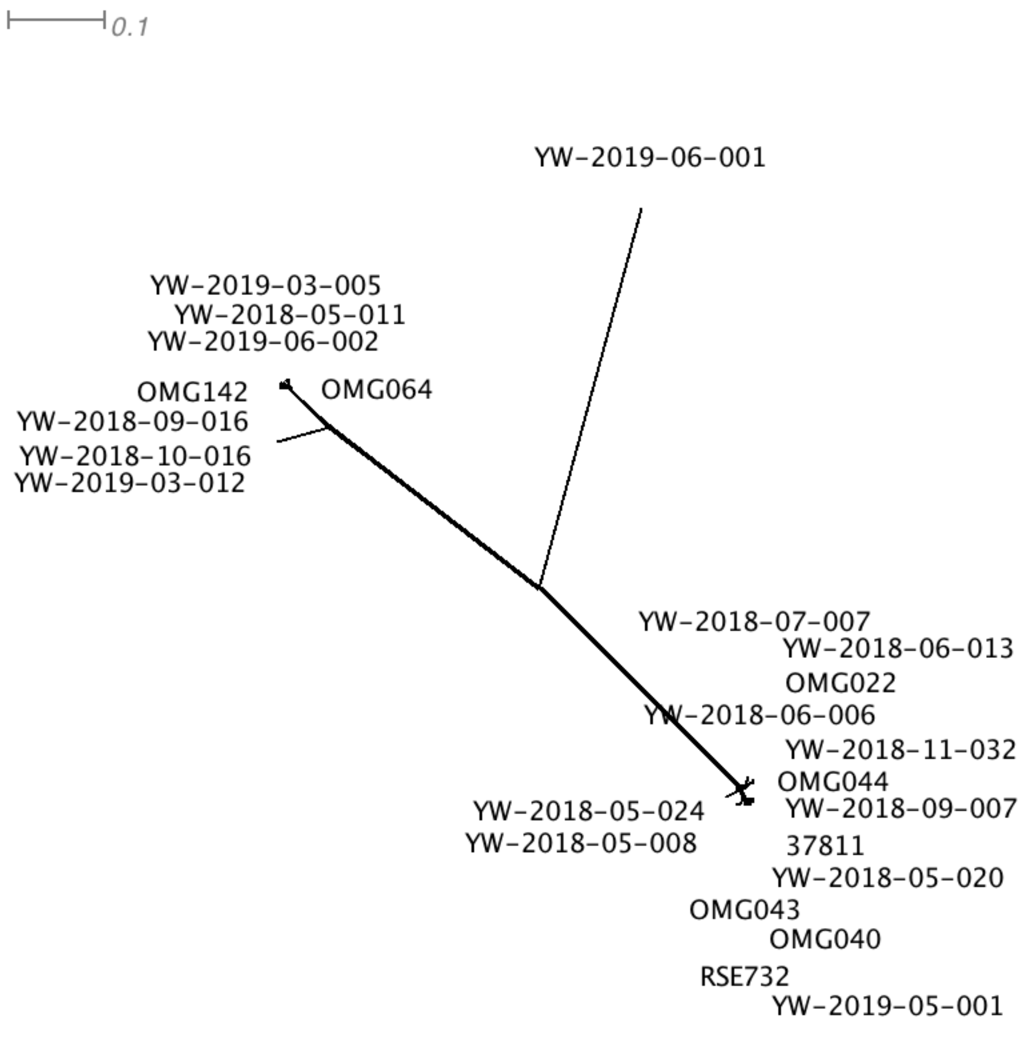

Locus #29

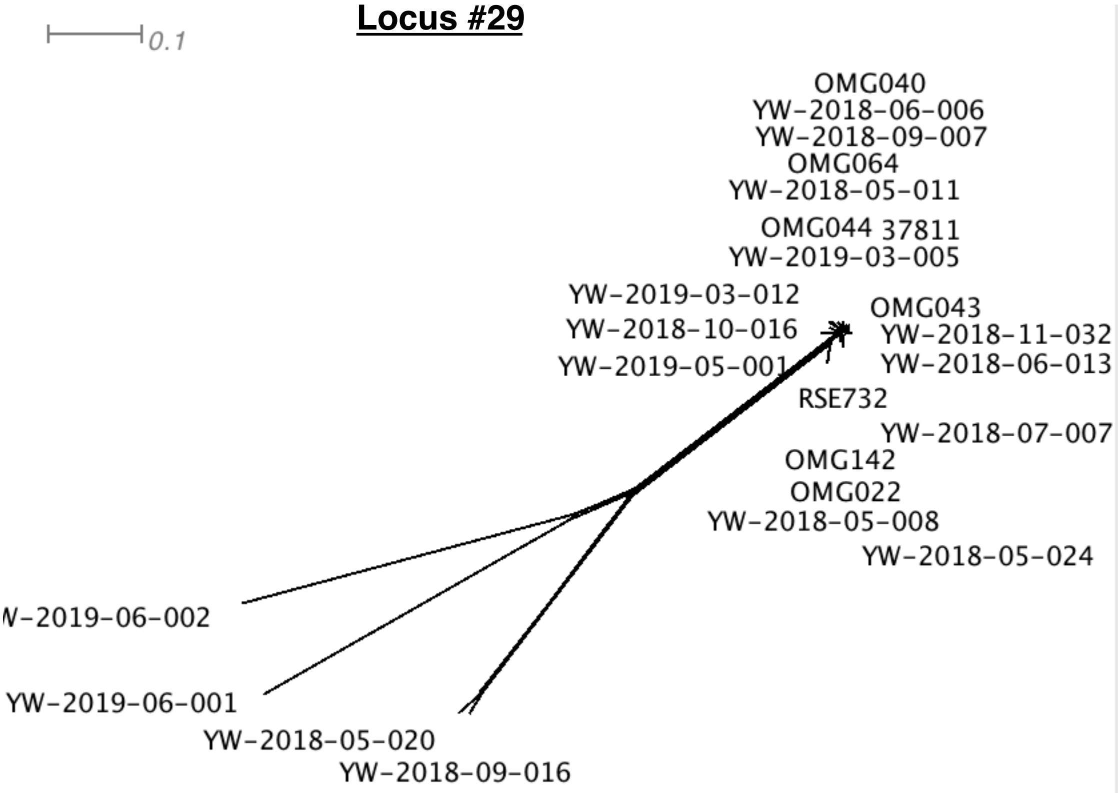
0.1
OMG040
YW-2018-06-006
YW-2018-09-007
OMG064
YW-2018-05-011
OMG044 37811
YW-2019-03-005
YW-2019-03-012
YW-2018-10-016
YW-2019-05-001
OMG043
YW-2018-11-032
YW-2018-06-013
RSE732
YW-2018-07-007
OMG142
OMG022
YW-2018-05-008
YW-2018-05-024
W-2019-06-002
YW-2019-06-001
YW-2018-05-020
YW-2018-09-016

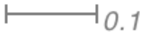
0.1

Locus #31

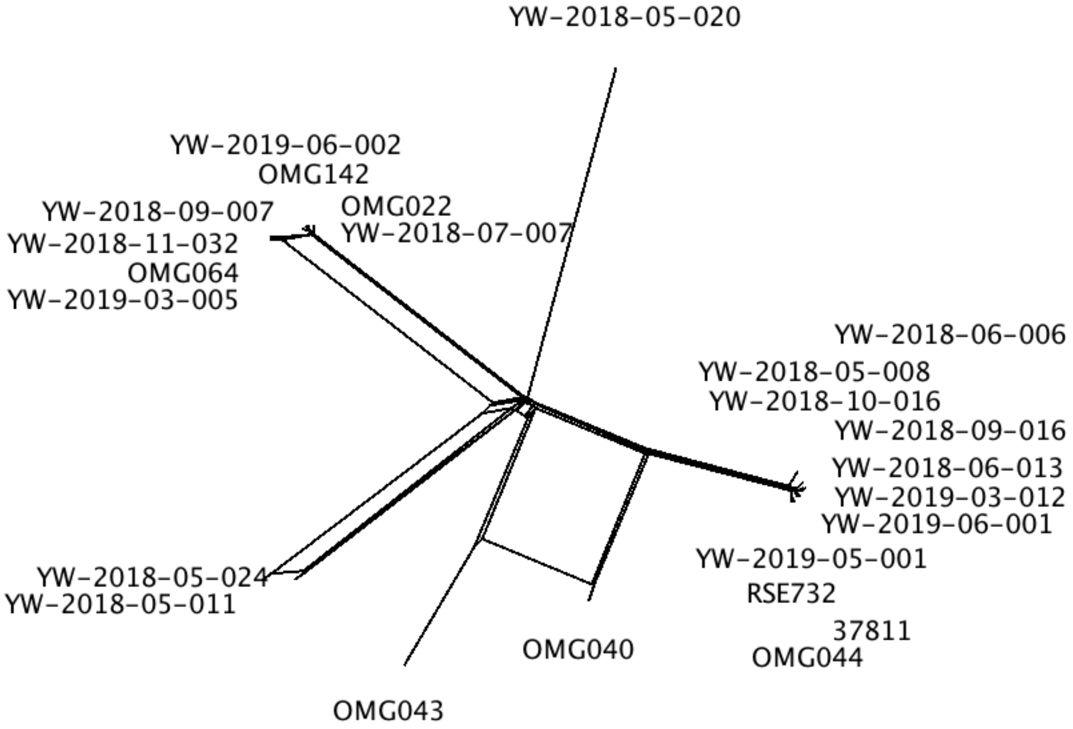
YW-2018-05-020
YW-2019-06-002
OMG142
YW-2018-09-007
OMG022
YW-2018-07-007
YW-2018-11-032
OMG064
YW-2019-03-005
YW-2018-06-006
YW-2018-05-008
YW-2018-10-016
YW-2018-09-016
YW-2018-06-013
YW-2019-03-012
YW-2019-06-001
YW-2019-05-001
RSE732
37811
OMG044
YW-2018-05-024
YW-2018-05-011
OMG040
OMG043

Locus #32

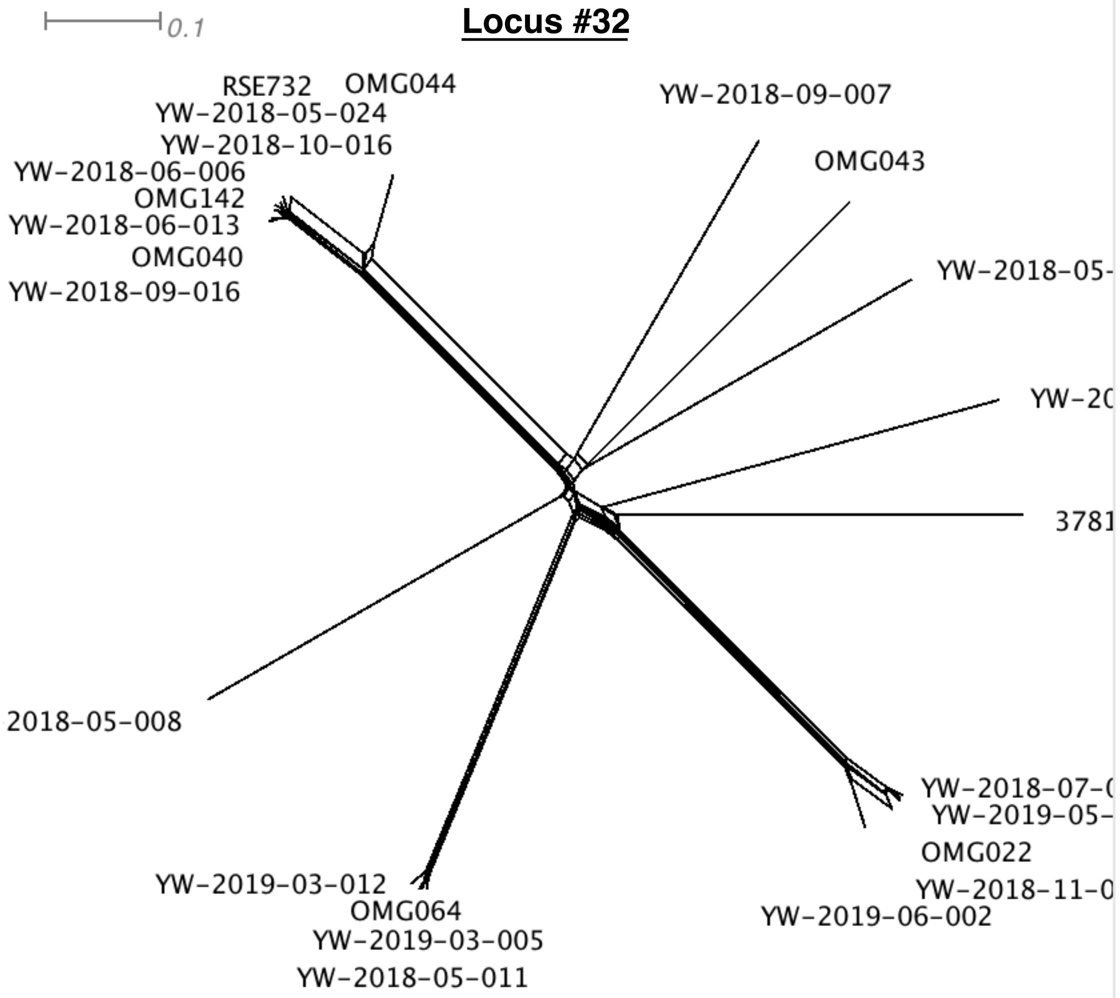
0.1
RSE732
OMG044
YW-2018-05-024
YW-2018-10-016
YW-2018-06-006
OMG142
YW-2018-06-013
OMG040
YW-2018-09-016
YW-2018-09-007
OMG043
YW-2018-05-
YW-20
3781
2018-05-008
YW-2018-07-
YW-2019-05-
OMG022
YW-2018-11-0
YW-2019-06-002
YW-2019-03-012
OMG064
YW-2019-03-005
YW-2018-05-011

Locus #103

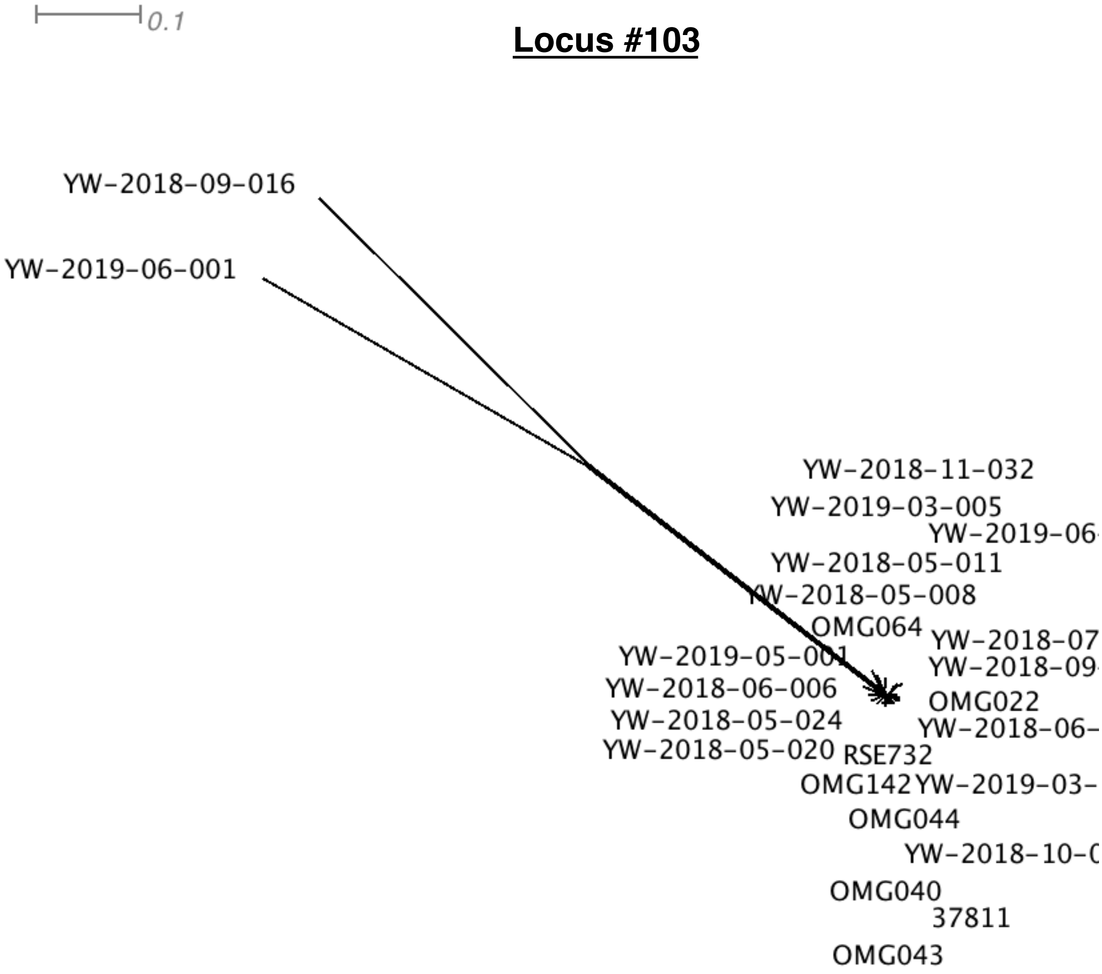
0.1
YW-2018-09-016
YW-2019-06-001
YW-2018-11-032
YW-2019-03-005
YW-2019-06
YW-2018-05-011
YW-2018-05-008
OMG064
YW-2018-07
YW-2019-05-001
YW-2018-09
YW-2018-06-006
OMG022
YW-2018-05-024
YW-2018-06-
YW-2018-05-020
RSE732
OMG142
YW-2019-03-
OMG044
YW-2018-10-
OMG040
37811
OMG043

Locus #113

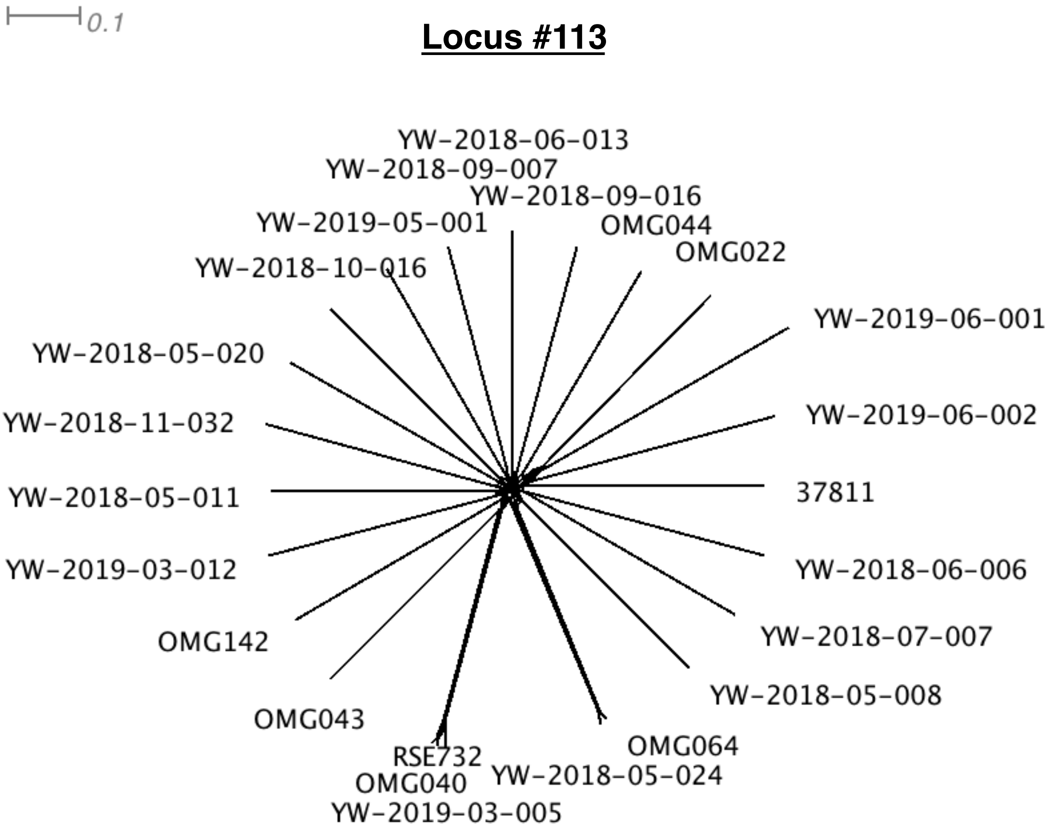
0.1
YW-2018-06-013
YW-2018-09-007
YW-2018-09-016
YW-2019-05-001
OMG044
OMG022
YW-2018-10-016
YW-2019-06-001
YW-2018-05-020
YW-2019-06-002
YW-2018-11-032
37811
YW-2018-05-011
YW-2019-03-012
YW-2018-06-006
YW-2018-07-007
OMG142
YW-2018-05-008
OMG043
OMG064
RSE732
OMG040
YW-2018-05-024
YW-2019-03-005

Locus #117

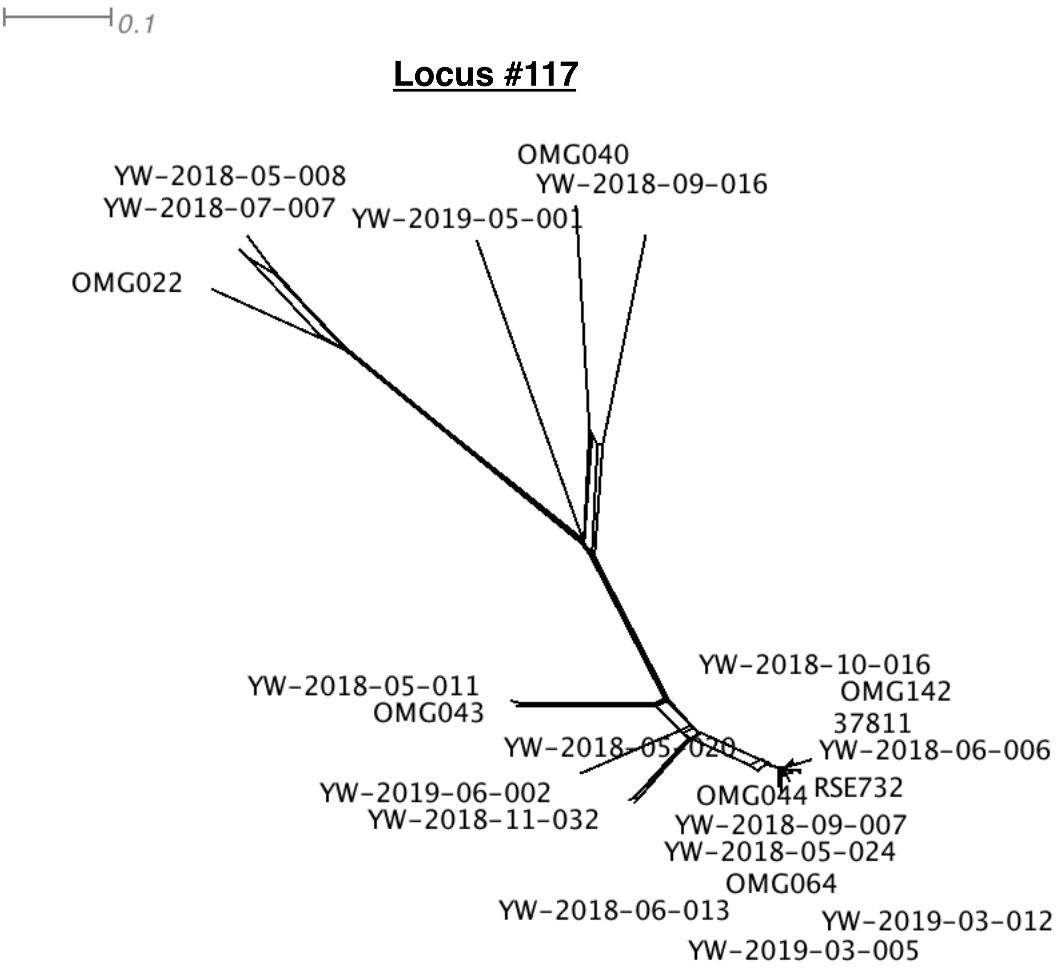
0.1
YW-2018-05-008
YW-2018-07-007
OMG022
OMG040
YW-2018-09-016
YW-2019-05-001
YW-2018-05-011
OMG043
YW-2018-10-016
OMG142
37811
YW-2018-06-006
YW-2018-05-020
RSE732
OMG044
YW-2019-06-002
YW-2018-11-032
YW-2018-09-007
YW-2018-05-024
OMG064
YW-2018-06-013
YW-2019-03-012
YW-2019-03-005

Locus #118

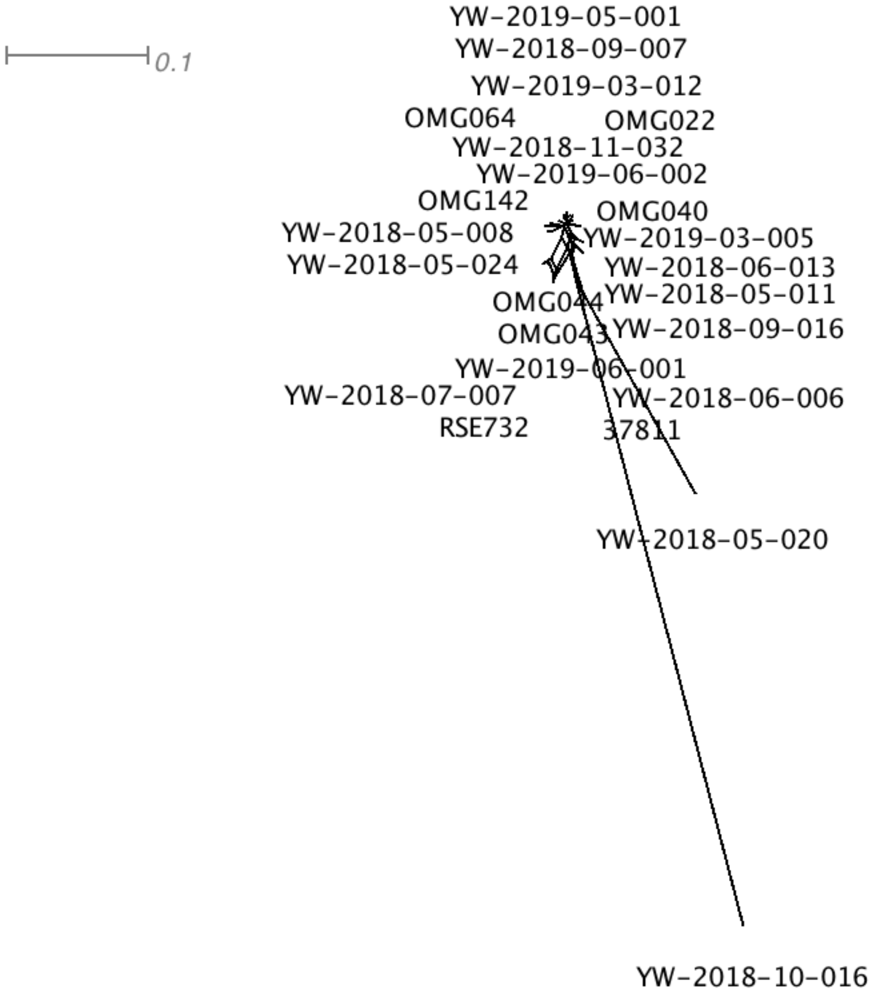
0.1
YW-2019-05-001
YW-2018-09-007
YW-2019-03-012
OMG064
OMG022
YW-2018-11-032
YW-2019-06-002
OMG142
OMG040
YW-2018-05-008
YW-2019-03-005
YW-2018-05-024
YW-2018-06-013
YW-2018-05-011
OMG044
OMG043
YW-2018-09-016
YW-2019-06-001
YW-2018-07-007
YW-2018-06-006
RSE732
37811
YW-2018-05-020
YW-2018-10-016

Locus #132

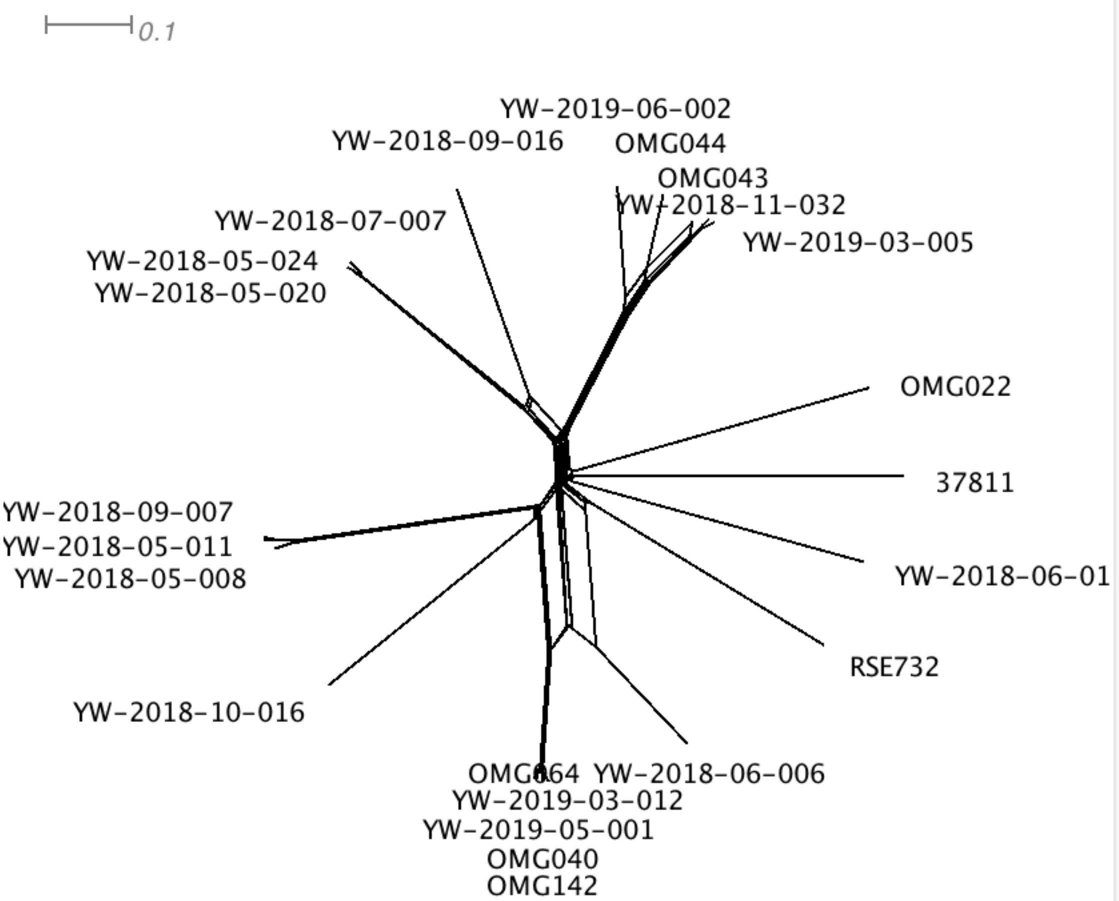
0.1
YW-2019-06-002
YW-2018-09-016
OMG044
OMG043
YW-2018-11-032
YW-2018-07-007
YW-2019-03-005
YW-2018-05-024
YW-2018-05-020
OMG022
37811
YW-2018-09-007
YW-2018-05-011
YW-2018-05-008
YW-2018-06-01
RSE732
YW-2018-10-016
OMG064
YW-2018-06-006
YW-2019-03-012
YW-2019-05-001
OMG040
OMG142

Locus #134

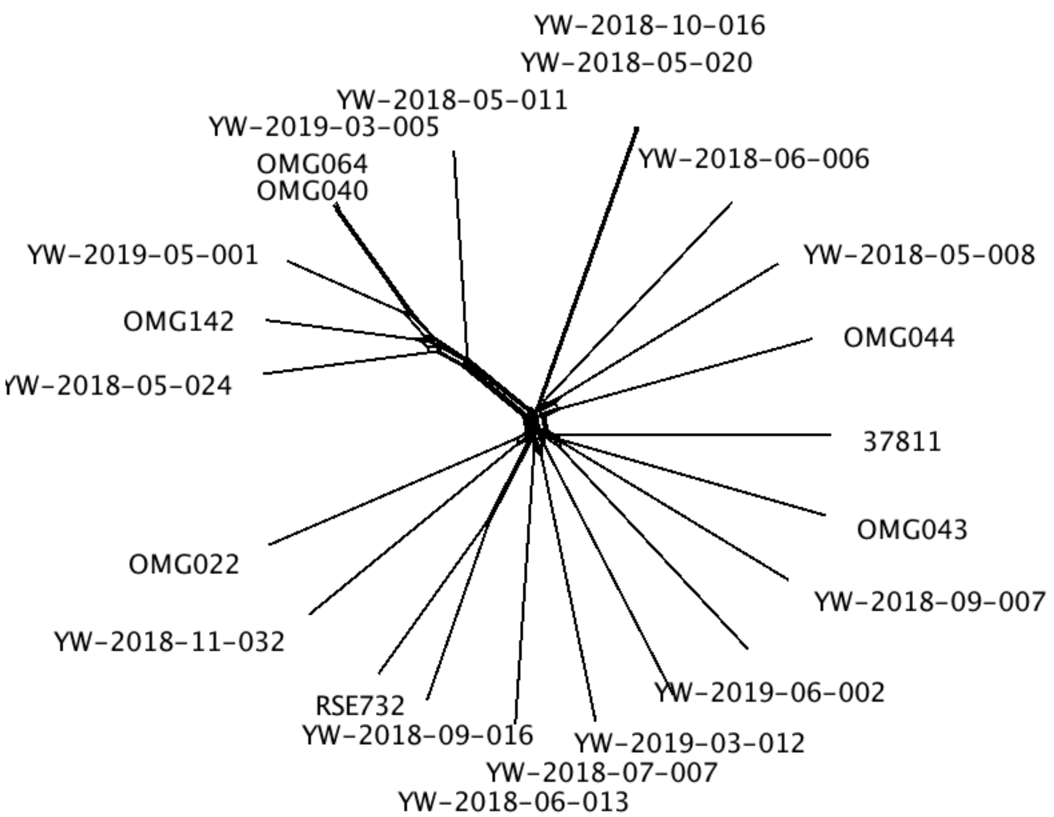
0.1
YW-2018-10-016
YW-2018-05-020
YW-2018-05-011
YW-2019-03-005
OMG064
OMG040
YW-2018-06-006
YW-2019-05-001
YW-2018-05-008
OMG142
OMG044
YW-2018-05-024
37811
OMG043
OMG022
YW-2018-09-007
YW-2018-11-032
YW-2019-06-002
RSE732
YW-2018-09-016
YW-2019-03-012
YW-2018-07-007
YW-2018-06-013

Locus #152

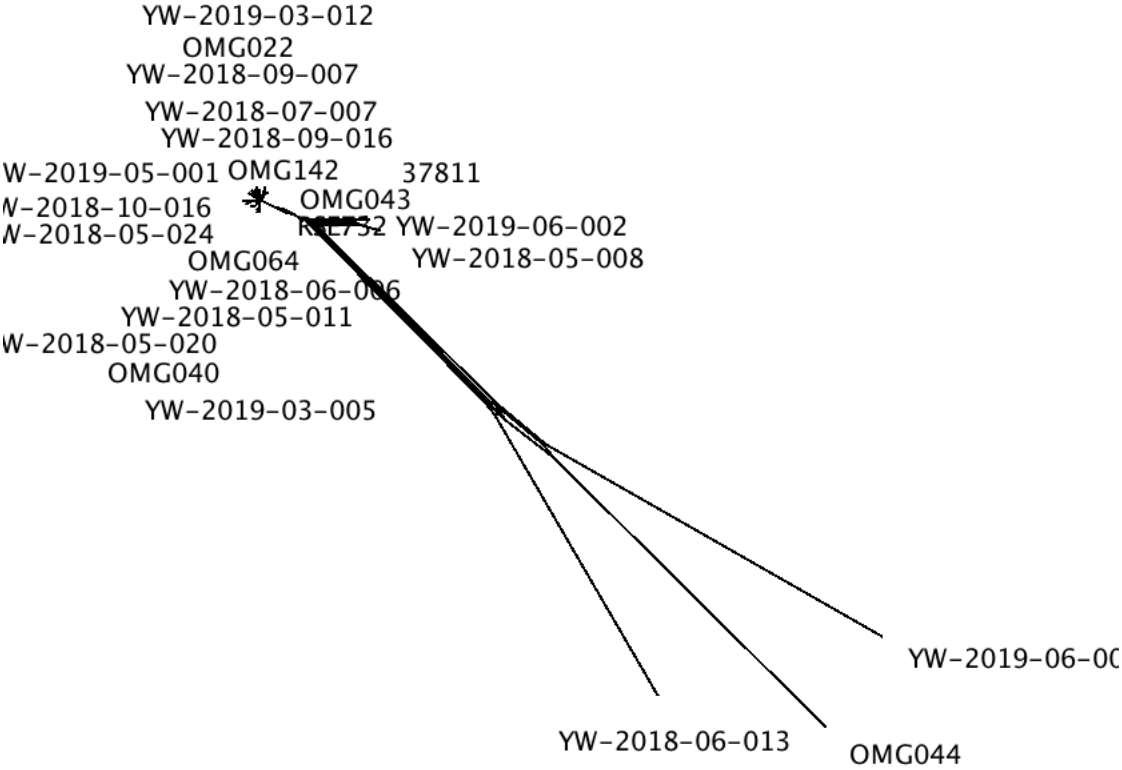

W-2018-11-032
YW-2019-03-012
OMG022
YW-2018-09-007
YW-2018-07-007
YW-2018-09-016
W-2019-05-001
OMG142
37811
OMG043
YW-2019-06-002
YW-2018-05-008
OMG064
YW-2018-05-011
W-2018-05-020
OMG040
YW-2019-03-005
YW-2019-06-00
YW-2018-06-013
OMG044

Locus #165

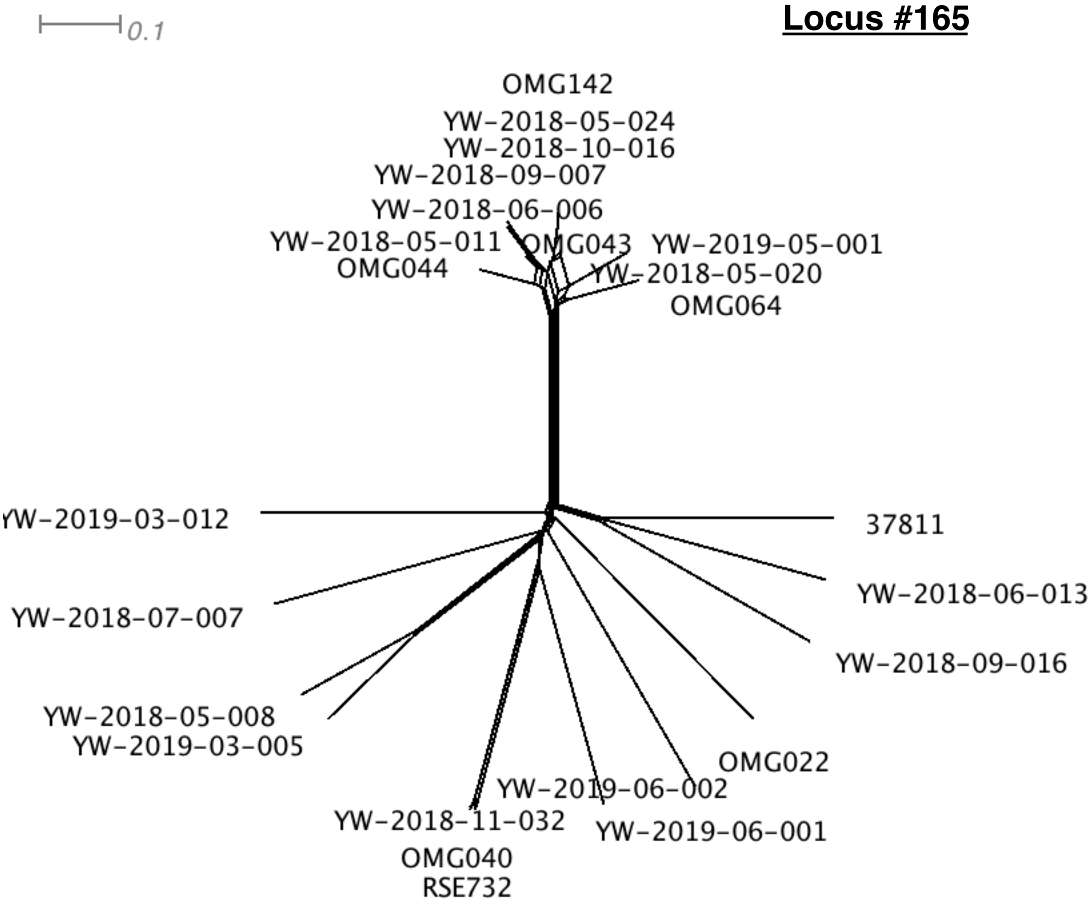
0.1
OMG142
YW-2018-05-024
YW-2018-10-016
YW-2018-09-007
YW-2018-06-006
YW-2018-05-011
OMG043
YW-2019-05-001
OMG044
YW-2018-05-020
OMG064
YW-2019-03-012
37811
YW-2018-06-013
YW-2018-07-007
YW-2018-09-016
YW-2018-05-008
YW-2019-03-005
OMG022
YW-2019-06-002
YW-2018-11-032
YW-2019-06-001
OMG040
RSE732
