# Supplementary figures and images for: A customised target capture sequencing tool for molecular identification of Aloe vera and relatives
Source: Sci Rep. 2021 Dec 21;11:24347. doi: 10.1038/s41598-021-03300-0 (PMC8692607; doi:10.1038/s41598-021-03300-0)

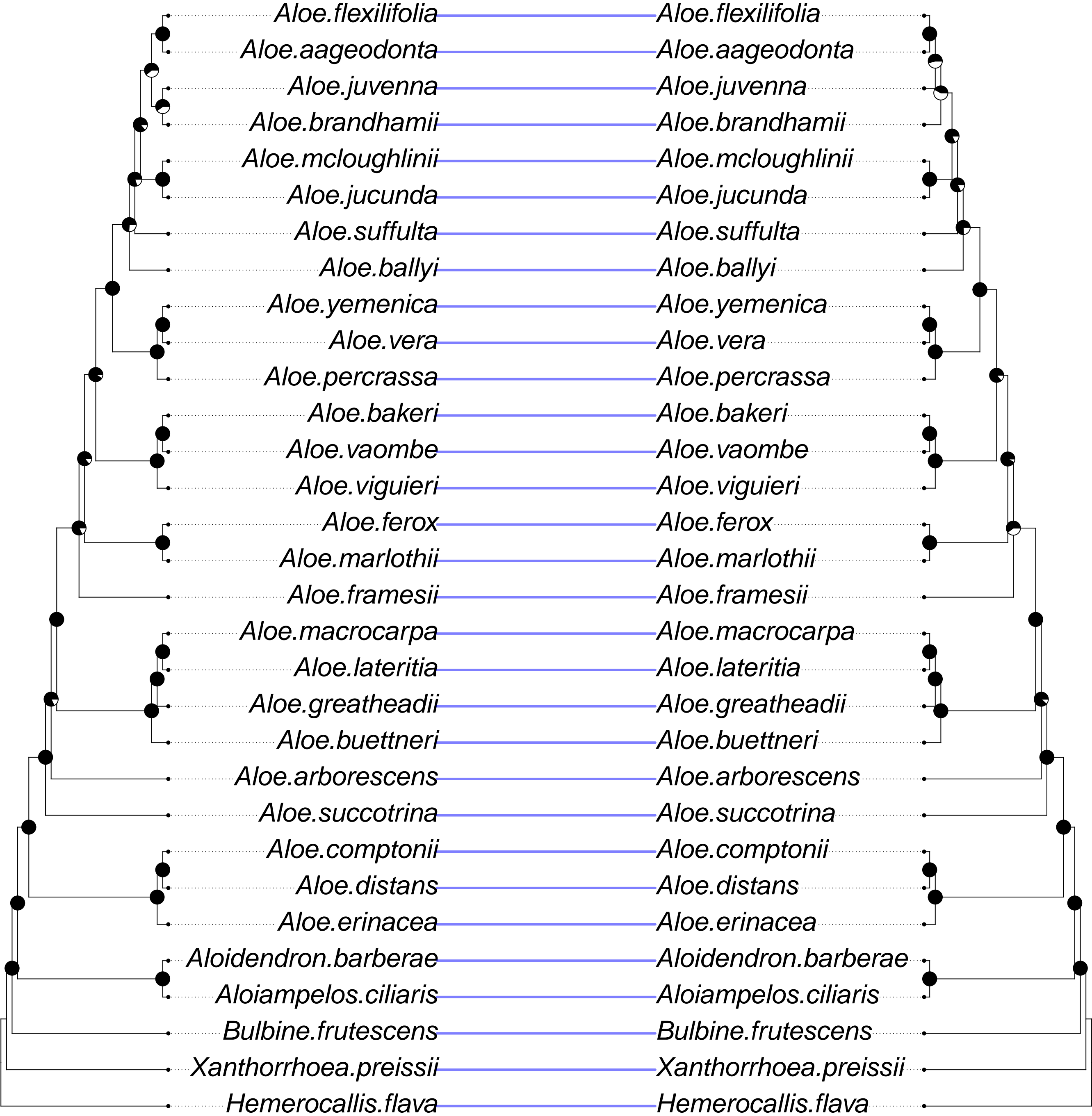

Supplement: Supplementary file 7 — Supplementary Information 7. [file 41598_2021_3300_MOESM7_ESM.pdf]
