# Supplementary material for: A customised target capture sequencing tool for molecular identification of Aloe vera and relatives
Source: Sci Rep. 2021 Dec 21;11:24347. doi: 10.1038/s41598-021-03300-0 (PMC8692607; doi:10.1038/s41598-021-03300-0)
Supplement: Supplementary file 9 — Supplementary Information 9. [file 41598_2021_3300_MOESM9_ESM.pdf]

E1: YW-2019-06-001

[SUPPLEMENTARY FILE S9]: Electrophoresis spectra for DNA extracts from museum specimens

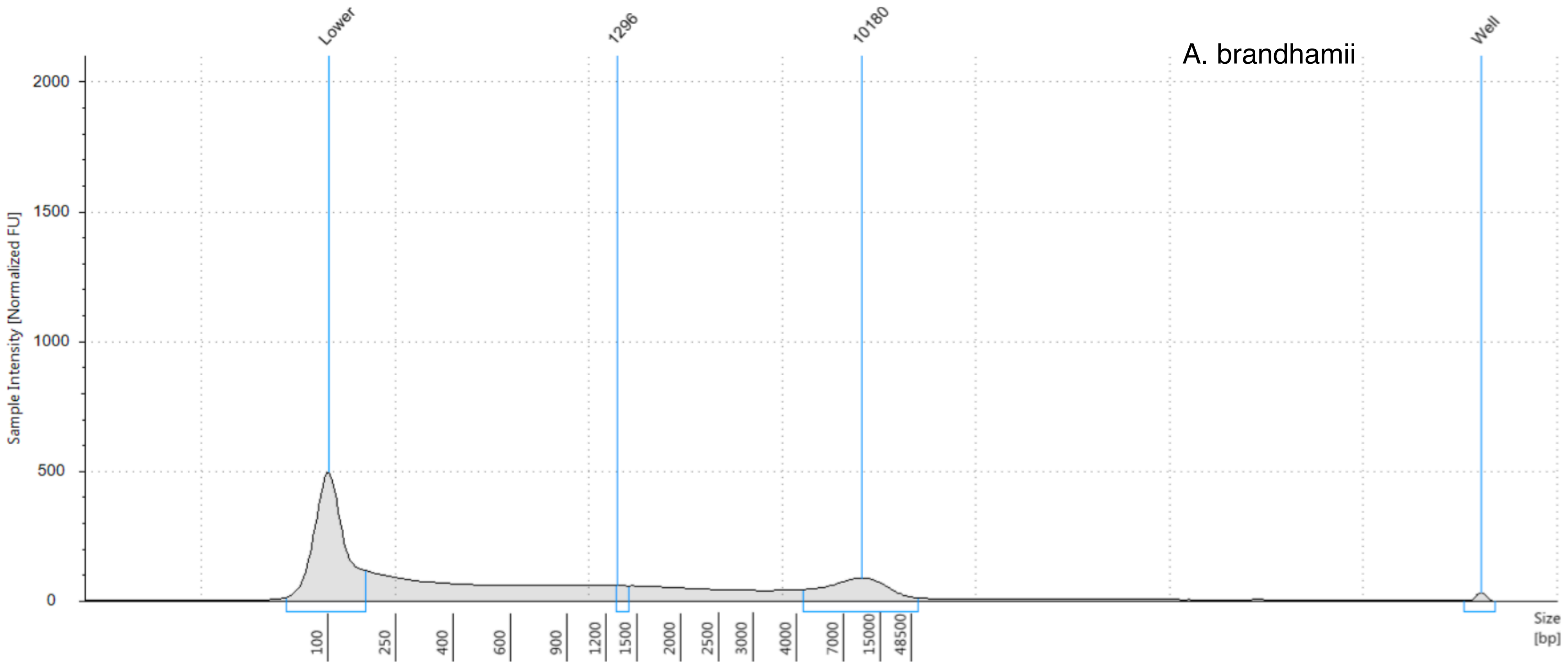

Sample Table

Well	DIN	Conc. [ng/µl]	Sample Description	Alert	Observations
E1	3.9	16.8	YW-2019-06-001	⚠	Caution! Expired ScreenTape device

Peak Table

Size [bp]	Calibrated Conc. [ng/µl]	Assigned Conc. [ng/µl]	% Integrated Area	From [bp]	To [bp]	Peak Comment	Observations
100	8.50	8.50	-	56	163		Lower Marker
1296	0.424	-	11.26	1283	1418		
10180	3.17	-	84.13	4279	55637		
-	-	-	-	-	-		Sample Well

F1: YW-2019-06-002

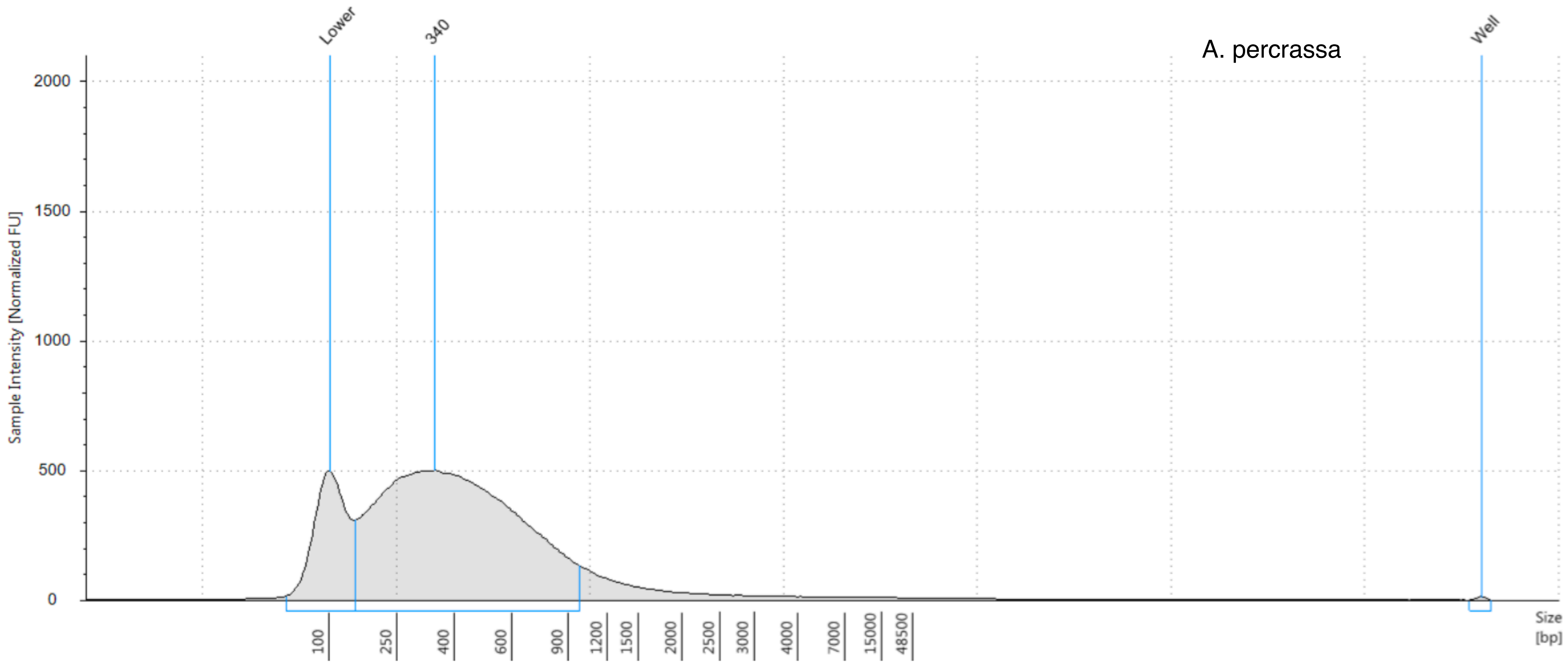

Sample Table

Well	DIN	Conc. [ng/µl]	Sample Description	Alert	Observations
F1	1.2	41.3	YW-2019-06-002	⚠	Caution! Expired ScreenTape device

Peak Table

Size [bp]	Calibrated Conc. [ng/µl]	Assigned Conc. [ng/µl]	% Integrated Area	From [bp]	To [bp]	Peak Comment	Observations
100	8.50	8.50	-	56	141		Lower Marker
340	36.8	-	99.86	141	977		
-	-	-	-	-	-		Sample Well
