# Supplementary material for: A customised target capture sequencing tool for molecular identification of Aloe vera and relatives
Source: Sci Rep. 2021 Dec 21;11:24347. doi: 10.1038/s41598-021-03300-0 (PMC8692607; doi:10.1038/s41598-021-03300-0)
Supplement: Supplementary file 10 — Supplementary Information 10. [file 41598_2021_3300_MOESM10_ESM.docx]

[TITLE]: “A customised target capture sequencing tool for molecular identification of Aloe vera and related species”

[SUPPLEMENTARY FILE S10]: Script for comparative phylogenomic analysis in R

##Script for plotting and comparing two ML-trees in a tanglegram

#Tree 1 is a Maximum-likelihood tree based on 189 low-copy nuclear genes (Aloe bait panel)

#Tree 2 is a Maximum-likelihood tree based on the "traditional barcoding loci" used in Grace et al 2015

#Load packages

library(ape)

library(ggplot2)

library(phytools)

#Importing tree 1

tr1 <- read.tree("/Volumes/Yannick_Woudstra_PhD_sequencing_files/Aloe_Pilot/analysis_phylogenetics-output/with-outgroup/Aloe_pilot_transcriptome_outgroup_concat.treefile")

plot(tr1)

#Finding the node which to reroot tree 1 from

findMRCA(tr1, tips=c("YW019","YW052"), type = "node")

#Rerooting tree 1

tr1.reroot <- ape::root(as.phylo(tr1), node=58, resolve.root=TRUE)

plot(tr1.reroot)

#Relabeling tree 1

tr1.reroot$tip.label

newlab <- c("Bulbine.frutescens","Aloe.jucunda","Aloe.mcloughlinii","Aloe.vera","Aloe.yemenica","Aloe.percrassa","Aloe.ballyi","Aloe.brandhamii","Aloe.juvenna","Aloe.flexilifolia","Aloe.aageodonta","Aloe.suffulta","Aloe.bakeri","Aloe.vaombe","Aloe.viguieri","Aloe.greatheadii","Aloe.macrocarpa","Aloe.lateritia","Aloe.ferox","Aloe.marlothii","Aloe.framesii","Aloe.arborescens","Aloe.succotrina","Aloe.comptonii","Aloe.distans","Aloe.erinacea","Aloidendron.barberae","Aloiampelos.ciliaris","Hemerocallis","Xanthorrhoea")

tr1.reroot$tip.label <- newlab

#Importing tree 2

tr2 <- read.tree("/Volumes/Yannick_Woudstra_PhD_sequencing_files/Aloe_Pilot/analysis_phylogenetics-output/with-outgroup/Grace2015_Pilot_outgroup_concat.treefile")

plot(tr2)

#Finding the node which to reroot tree 2 from

findMRCA(tr2, tips=c("Hemerocallis","Xanthorrhoea"), type = "node")

#Rerooting tree 2

tr2.reroot <- ape::root(as.phylo(tr2), node=49, resolve.root=TRUE)

plot(tr2.reroot)

#This is to remove branch lengths

tr1.reroot$edge.length<-NULL

tr2.reroot$edge.length<-NULL

#Adding missing support values (tr2, 90 for node between Aloe juvenna and Aloe aageodonta; tr1, 100 for node between Bulbine and the rest)

tr2.reroot$node.label

tr2.reroot$node.label[[2]] <- "90"

tr1.reroot$node.label

tr1.reroot$node.label[[2]] <- "100"

#Making tanglegrams

tang1 <- cophylo(tr1.reroot, tr2.reroot)

pdf("/Volumes/macOShdA11888/Users/yw13kg/Desktop/Aloe_pilot_tanglegram.pdf", width = 30, height = 15)

plot(tang1, link.type="curved", link.lwd=3,link.lty="solid",link.col=make.transparent("blue",0.5),cex=1,fsize=2)

#Adding support values to the trees, as pie charts!!

nodelabels.cophylo(node=1:tang1$trees[[1]]$Nnode+Ntip(tang1$trees[[1]]),

pie=cbind(as.numeric(tang1$trees[[1]]$node.label),100-as.numeric(tang1$trees[[1]]$node.label)),

piecol=c("black","white"),cex=0.3,which="left")

nodelabels.cophylo(node=1:tang1$trees[[2]]$Nnode+Ntip(tang1$trees[[2]]),

pie=cbind(as.numeric(tang1$trees[[2]]$node.label),100-as.numeric(tang1$trees[[2]]$node.label)),

piecol=c("black","white"),cex=0.3,which="right")

dev.off()
