# Supplementary material for: A customised target capture sequencing tool for molecular identification of Aloe vera and relatives
Source: Sci Rep. 2021 Dec 21;11:24347. doi: 10.1038/s41598-021-03300-0 (PMC8692607; doi:10.1038/s41598-021-03300-0)
Supplement: Supplementary file 11 — Supplementary Information 11. [file 41598_2021_3300_MOESM11_ESM.docx]

[TITLE]: “A customised target capture sequencing tool for molecular identification of Aloe vera and related species”

[SUPPLEMENTARY FILE S11]: Script for phylogenetic analysis of ASTRAL results in R

#Script for plotting the ASTRAL tree for the full dataset used in the Aloe bait panel pilot study

#Tree is a summary of 189 ML-gene trees calculated with IQtree

#It seems from the ASTRAL results that one gene tree was dropped after removing branches with <10 BS support

#Load packages

library(ape)

library(ggplot2)

library(phytools)

#Importing the tree

tree1 <- read.tree("/Volumes/Yannick_Woudstra_PhD_sequencing_files/Aloe_Pilot/analysis_phylogenetics-output/with-outgroup/Aloe_pilot_all-loci_outgroup_iqtree_BS10_astral_scored.tre")

plot(tree1)

#Using the proper outgroup improved the rooting of the tree as the proper order of lineages was retrieved here.

#Rerooting is therefore not necessary.

#Tree tips need to be relabeled

tree1$tip.label

newlab <- c("Hemerocallis.flava","Xanthorrhoea.preissii","Bulbine.frutescens","Aloiampelos.ciliaris","Aloidendron.barberae","Aloe.erinacea","Aloe.comptonii","Aloe.distans","Aloe.succotrina","Aloe.arborescens","Aloe.buettneri","Aloe.greatheadii","Aloe.lateritia","Aloe.macrocarpa","Aloe.framesii","Aloe.marlothii","Aloe.ferox","Aloe.viguieri","Aloe.vaombe","Aloe.bakeri","Aloe.percrassa","Aloe.vera","Aloe.yemenica","Aloe.ballyi","Aloe.suffulta","Aloe.juvenna","Aloe.brandhamii","Aloe.aageodonta","Aloe.flexilifolia","Aloe.jucunda","Aloe.mcloughlinii")

tree1$tip.label <- newlab

pdf("/Volumes/macOShdA11888/Users/yw13kg/Desktop/Aloe_pilot_ASTRAL.pdf", width = 15, height = 15)

plot(tree1, cex=2)

nodelabels(node=1:tree1$Nnode+Ntip(tree1), pie =cbind(as.numeric(tree1$node.label),1-as.numeric(tree1$node.label)),piecol=c("black","white"),cex=0.5)

dev.off()

tree1$node.label

#tree2 was constructed with orthologs only (177 loci)

tree2 <- read.tree("/Volumes/Yannick_Woudstra_PhD_sequencing_files/Aloe_Pilot/analysis_phylogenetics-output/with-outgroup/Aloe_pilot_orthologs_outgroup_iqtree_BS10_astral_scored.tre")

plot(tree2)

tree2$tip.label

newlab2 <- c("Hemerocallis.flava","Xanthorrhoea.preissii","Bulbine.frutescens","Aloiampelos.ciliaris","Aloidendron.barberae","Aloe.erinacea","Aloe.distans","Aloe.comptonii","Aloe.succotrina","Aloe.arborescens","Aloe.buettneri","Aloe.greatheadii","Aloe.lateritia","Aloe.macrocarpa","Aloe.framesii","Aloe.marlothii","Aloe.ferox","Aloe.viguieri","Aloe.vaombe","Aloe.bakeri","Aloe.percrassa","Aloe.vera","Aloe.yemenica","Aloe.ballyi","Aloe.suffulta","Aloe.jucunda","Aloe.mcloughlinii","Aloe.brandhamii","Aloe.juvenna","Aloe.aageodonta","Aloe.flexilifolia")

tree2$tip.label <- newlab2

tree1$edge.length<-NULL

tree2$edge.length<-NULL

tang2 <- cophylo(tree1, tree2)

pdf("/Volumes/macOShdA11888/Users/yw13kg/Desktop/Aloe_pilot_ASTRAL_tanglegram.pdf", width = 15, height = 15)

plot(tang2, link.type="curved", link.lwd=3,link.lty="solid",link.col=make.transparent("blue",0.5),cex=1,fsize=2)

nodelabels.cophylo(node=1:tang2$trees[[1]]$Nnode+Ntip(tang2$trees[[1]]),

pie=cbind(as.numeric(tang2$trees[[1]]$node.label),1-as.numeric(tang2$trees[[1]]$node.label)),

piecol=c("black","white"),cex=0.3,which="left")

nodelabels.cophylo(node=1:tang2$trees[[2]]$Nnode+Ntip(tang2$trees[[2]]),

pie=cbind(as.numeric(tang2$trees[[2]]$node.label),1-as.numeric(tang2$trees[[2]]$node.label)),

piecol=c("black","white"),cex=0.3,which="right")

dev.off()
