# Supplementary material for: A customised target capture sequencing tool for molecular identification of Aloe vera and relatives
Source: Sci Rep. 2021 Dec 21;11:24347. doi: 10.1038/s41598-021-03300-0 (PMC8692607; doi:10.1038/s41598-021-03300-0)
Supplement: Supplementary file 12 — Supplementary Information 12. [file 41598_2021_3300_MOESM12_ESM.docx]

[TITLE]: **A customised target capture sequencing tool for molecular identification of *Aloe vera* and relatives**

Yannick Woudstra^1,2^*, Juan Viruel^1^, Martin Fritzsche^3^, Thomas Bleazard^3^, Ryan Mate^3^, Caroline Howard^4^, Nina Rønsted^2,5^, Olwen M. Grace^1^

^1^ Royal Botanic Gardens, Kew, Surrey TW9 3AE, United Kingdom

^2^ Natural History Museum Denmark, University of Copenhagen, Gothersgade 130, Copenhagen 1153, Denmark

^3^ National Institute of Biological Standards and Control, South Mimms, United Kingdom

^4^ Wellcome Sanger Institute, Wellcome Trust Genome Campus, Hinxton, Saffron Walden CB10 1RQ, United Kingdom

^5^ National Tropical Botanical Garden, 3530 Papalina Road, Kalaheo, HI 96741, USA

*Corresponding author: yannickwoudstra@outlook.com; Twitter: @ywoudstra

**Supplementary Information**

[SUPPLEMENTARY FILE S1]: Transcriptome assembly statistics

Legend: Assembly statistics for each transcriptome sample. AAR = Aloe arborescens, ABA = Aloidendron Barberae, ABU = Aloe buettneri and AVE = Aloe vera. ^Read pairs after trimming. *Aloe vera transcriptome sequenced and assembled as part of the 1000 Plants Initiative project^1^ using SOAPdenovo assembly software. **Total number of contigs as determined from the available data. N50 = assembly quality as contiguity, sequence length of shortest contig at 50% of total transcript length.

[SUPPLEMENTARY FILE S2]: Gene annotation and statistics per locus

Legend: Detailed information on low-copy nuclear loci targeted by the designed bait panel. Columns B-E contain information determined using the sequences obtained from the transcriptomes, aligned to the *Oriza sativa* genome^2^ using MarkerMiner v1.2^3^. Columns F and G contain the ID numbers of overlapping loci in the Angiosperms-353^4^ and Angiosperm-V1^5^ tools, respectively. Columns H-Q contain information determined using the pilot study sequencing data, described in this study.

[SUPPLEMENTARY FILE S3]: Read coverage statistics pilot study

Legend: Details of read depth in the pilot study sequencing experiment using MiSeq (2x 300bp) for the ingroup samples and HiSeq (2x 150bp) for the outgroup samples. Sheet 1 contains read counts for each locus in all samples, calculated using indexed .bam files obtained from the HybPiper^6^ assembly pipeline and visualised in Tablet^7^. Sheet 2 provides the calculated read depth, as described in section 5.3. Minimum, maximum and average read depth were calculated for each locus and for the full target dataset, which was done separately for ingroup and outgroup samples.

[SUPPLEMENTARY FILE S4]: Recovery comparison with Angiosperms-353 bait panel

Legend: Comparison of recovered target gene length and percentage of target gene for overlapping loci between the customised *Aloe* bait panel and the universal Angiosperms-353^4^ bait panel. Overlapping loci were identified by performing a local BLAST in Geneious® PRO using the Angiosperms-353 panel as query and the genes from the *Aloe* bait panel as subject. Recovery statistics for Alooideae taxa using the Angiosperms-353 panel were obtained from unpublished work (Grace et al., in review).

[SUPPLEMENTARY FILE S5]: Traditional loci sequence accessions

Legend: Sequence accessions on NCBI for traditional loci sequences in the phylogenetic comparison experiment (section 5.5). Sequence accessions noted in italicised text are sequences generated in this study, as described in section 5.5, other sequence accessions were obtained from NCBI and were generated by previous studies^8,9^.

[SUPPLEMENTARY FILE S6]: SplitsTree trees for paralogy indication

Legend: Displays of unrooted gene trees produced using SplitsTree v4.16^10^ for the twelve loci for which paralogy was confirmed. The confirmation comes from the presence of a clear bi- (or multi-) modal segregation of taxa along with long branches separating the clades in the unrooted tree.

[SUPPLEMENTARY FILE S7]: Cophylogeny comparing full LCN dataset and reduced (ortholog-only) dataset

Legend: Two maximum likelihood trees representing the full LCN dataset (left) and the reduced dataset with paralogs removed (right), aligned together to indicated differences in topology. Trees are produced from a concatenated dataset using IQTree v1.6.12^11^ and aligned using the R-package phytools v0.7-70^12^. Pie charts on nodes indicate bootstrap support, calculated with 1000 replicates, with full pies (black) indicating full support (=100).

[SUPPLEMENTARY FILE S8]: Accession information

Legend: Details of provenance and sample information on the plant accessions used in this study.

[SUPPLEMENTARY FILE S9]: Electrophoresis spectra for DNA extracts from museum specimens

Legend: Electrophoresis spectra obtain with an Agilent 4200 TapeStation indicating the distribution of DNA fragment length in the two herbarium samples used in this study.

[SUPPLEMENTARY FILE S10]: Script for comparative phylogenomic analysis in R

Legend: Script for code used in R to produce the figures used in the comparative phylogenomic analysis (section 5.5).

[SUPPLEMENTARY FILE S11]: Script for phylogenetic analysis of ASTRAL results in R

Legend: Script for code used in R to produce the figures used in the phylogenetic analysis of the LCN dataset using a coalescent-based model with ASTRAL-III^13^.

**References**

1 Carpenter, E. J. *et al.* Access to RNA-sequencing data from 1,173 plant species: The 1000 Plant transcriptomes initiative (1KP). *GigaScience* **8**, doi:10.1093/gigascience/giz126 (2019).

2 Ouyang, S. *et al.* The TIGR Rice Genome Annotation Resource: improvements and new features. *Nucleic Acids Research* **35**, D883-D887, doi:10.1093/nar/gkl976 (2006).

3 Chamala, S. *et al.* MarkerMiner 1.0: A new application for phylogenetic marker development using angiosperm transcriptomes. *Applications in plant sciences* **3**, apps.1400115, doi:10.3732/apps.1400115 (2015).

4 Johnson, M. G. *et al.* A Universal Probe Set for Targeted Sequencing of 353 Nuclear Genes from Any Flowering Plant Designed Using k-Medoids Clustering. *Systematic Biology* **68**, 594-606, doi:10.1093/sysbio/syy086 (2018).

5 Buddenhagen, C. *et al.* Anchored Phylogenomics of Angiosperms I: Assessing the Robustness of Phylogenetic Estimates. *bioRxiv*, 086298, doi:10.1101/086298 (2016).

6 Johnson, M. G. *et al.* HybPiper: Extracting Coding Sequence and Introns for Phylogenetics from High-Throughput Sequencing Reads Using Target Enrichment. *Applications in Plant Sciences* **4** (2016).

7 Milne, I. *et al.* Using Tablet for visual exploration of second-generation sequencing data. *Briefings in Bioinformatics* **14**, 193-202, doi:10.1093/bib/bbs012 (2012).

8 Grace, O. M. *et al.* Evolutionary history and leaf succulence as explanations for medicinal use in aloes and the global popularity of Aloe vera. *BMC Evolutionary Biology* **15**, 29, doi:10.1186/s12862-015-0291-7 (2015).

9 Dee, R., Malakasi, P., Rakotoarisoa, S. E. & Grace, O. M. A phylogenetic analysis of the genus Aloe (Asphodelaceae) in Madagascar and the Mascarene Islands. *Botanical Journal of the Linnean Society* **187**, 428-440, doi:10.1093/botlinnean/boy026 (2018).

10 Huson, D. H. & Bryant, D. Application of Phylogenetic Networks in Evolutionary Studies. *Molecular Biology and Evolution* **23**, 254-267, doi:10.1093/molbev/msj030 (2005).

11 Nguyen, L.-T., Schmidt, H. A., von Haeseler, A. & Minh, B. Q. IQ-TREE: A Fast and Effective Stochastic Algorithm for Estimating Maximum-Likelihood Phylogenies. *Molecular Biology and Evolution* **32**, 268-274, doi:10.1093/molbev/msu300 (2014).

12 Revell, L. J. phytools: an R package for phylogenetic comparative biology (and other things). *Methods in Ecology and Evolution* **3**, 217-223, doi:https://doi.org/10.1111/j.2041-210X.2011.00169.x (2012).

13 Zhang, C., Rabiee, M., Sayyari, E. & Mirarab, S. ASTRAL-III: polynomial time species tree reconstruction from partially resolved gene trees. *BMC Bioinformatics* **19**, 153, doi:10.1186/s12859-018-2129-y (2018).
